# Understanding the origins of metal–organic framework/polymer compatibility†
†Electronic supplementary information (ESI) available: Experimental and computational details. See DOI: 10.1039/c7sc04152g


**DOI:** 10.1039/c7sc04152g

**Published:** 2017-10-27

**Authors:** R. Semino, J. C. Moreton, N. A. Ramsahye, S. M. Cohen, G. Maurin

**Affiliations:** a Institut Charles Gerhardt Montpellier UMR 5253 CNRS , Université de Montpellier , Place E. Bataillon , 34095 Montpellier Cedex 05 , France . Email: guillaume.maurin@univ-montp2.fr; b Department of Chemistry and Biochemistry , University of California , La Jolla , San Diego , California 92093-0358 , USA . Email: scohen@ucsd.edu; c Institut Charles Gerhardt Montpellier , UMR 5253 CNRS, UM, ENSCM , 8 rue de l’Ecole Normale , 34296 Montpellier Cedex 05 , France

## Abstract

The microscopic interfacial structures for a series of metal–organic framework/polymer composites consisting of the Zr-based UiO-66 coupled with different polymers are systematically explored by applying a computational methodology that integrates density functional theory calculations and force field-based molecular dynamics simulations. These predictions are correlated with experimental findings to unravel the structure–compatibility relationship of the MOF/polymer pairs. The relative contributions of the intermolecular MOF/polymer interactions and the flexibility/rigidity of the polymer with respect to the microscopic structure of the interface are rationalized, and their impact on the compatibility of the two components in the resulting composite is discussed. The most compatible pairs among those investigated involve more flexible polymers, *i.e.* polyvinylidene fluoride (PVDF) and polyethylene glycol (PEG). These polymers exhibit an enhanced contact surface, due to a better adaptation of their configuration to the MOF surface. In these cases, the irregularities at the MOF surface are filled by the polymer, and even some penetration of the terminal groups of the polymer into the pores of the MOF can be observed. As a result, the affinity between the MOF and the polymer is very high; however, the pores of the MOF may be sterically blocked due to the strong MOF/polymer interactions, as evidenced by UiO-66/PEG composites. In contrast, composites involving polymers that exhibit higher rigidity, such as the polymer of intrinsic microporosity-1 (PIM-1) or polystyrene (PS), present interfacial microvoids that contribute to a decrease in the contact surface between the two components, thus reducing the MOF/polymer affinity.

## Abstract 2

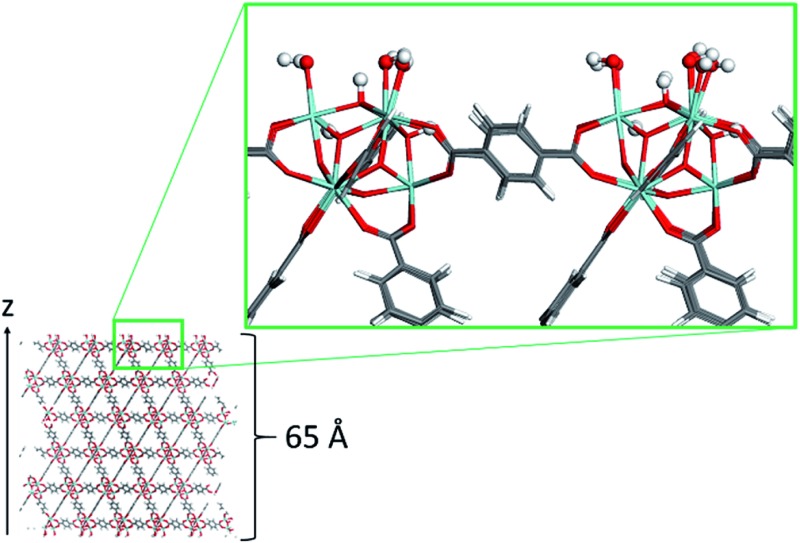
The microscopic interfacial structures for a series of metal–organic frameworks (MOFs)/polymer composites consisting of the Zr-based UiO-66 coupled with different polymers are systematically explored by applying a computational methodology that integrates density functional theory calculations and force field-based molecular dynamics simulations.

## Introduction

Mixed matrix membranes (MMMs) comprising polymers and metal–organic frameworks (MOFs) as fillers can enhance the performance of polymers as single components for separating challenging gas^1^–^4^/liquid^5^ mixtures, while maintaining easy processability.^6^ Moreover, it has recently been shown that these MMMs are promising candidates as proton exchange membranes.^7^,^8^ One of the main challenges to overcome in the field of MOF-based MMMs is that MOF/polymer compatibility is generally poor, and as a result, only low filler loading can be achieved before membrane selectivity is negatively affected.^9^ Furthermore, challenges remain toward fabricating uniform and defect-free MMMs that allow for homogenous dispersion of the MOFs in the polymer to avoid agglomeration and other defects.^10^,^11^ Therefore, understanding what makes a MOF/polymer pair compatible is essential to advance the development and utility of these composites. To this end, the microscopic origins of the MOF/polymer compatibility must be investigated.

A useful example of a MOF-derived MMM is that of ZIF-8 12 /polymer of intrinsic microporosity-1 13 (PIM-1) composite. PIM-1 has an unusually high permeability due to its rigidity that prevents an efficient packing of the polymer chains,^13^ while ZIF-8 is known to be highly selective for several gas separations.^14^–^17^ Therefore, their combination might be expected to benefit both from the permeability of PIM-1 and the selectivity of ZIF-8.^18^ Bushell *et al.* have synthesized MMMs based on this composite and studied their performances for CO_2_ capture over N_2_.^18^ They found that both CO_2_ permeability and CO_2_/N_2_ selectivity of the MMM increase with ZIF-8 loading. The increase in the permeability was suspected to be due to a global increase of the free volume of the system resulting from the cumulative porosity of the two components and additional voids created at the boundary between ZIF-8 nanoparticles and the PIM-1 matrix. We recently developed a methodology to model MOF/polymer interfaces by molecular simulations, and applied it to study the ZIF-8/PIM-1 case to shed light on the organization of the composites at the molecular level.^19^ The microscopic structure of the interface was characterized in terms of MOF/polymer interaction sites, as well as polymer rigidity and conformation at the interface. Well-defined independent microscopic voids were evidenced at the interface supporting the hypotheses developed from the experimental findings.^18^ In another study, ZIF-8/PIM-1 colloidal suspensions and membranes were prepared and carefully characterized by advanced experimental tools combined with molecular simulations.^20^ This study suggested that the microscopic structure of the composites depends on many factors including the physicochemical properties of the polymer and MOF/polymer interactions. Through these pioneering studies, the molecular reasons for the relatively poor compatibility of the ZIF-8/PIM-1 composites were unveiled.

In contrast, no reports are available that identify the microscopic origins for systems that demonstrate more favorable MOF/polymer compatibility. We have reported on the preparation of MMMs with a high MOF content that can be delaminated and are mechanically stable and pliable,^21^ which suggest excellent MOF/polymer compatibility. Notably, these membranes were shown to retain the high surface areas of the MOFs. Among the systems studied was a UiO-66/poly(vinylidene fluoride) (PVDF) composite.^22^ Here, we apply a computational approach to explain the microscopic origin of the improved compatibility in UiO-66/PVDF MMMs. The behavior of this composite is further compared with that predicted for three different UiO-66-based MMMs: using PIM-1, polystyrene (PS), and polyethylene glycol (PEG) as the polymer component. Composites including UiO-66 nanoparticles in PS,^23^ and in PIM-1 24,25 have already been reported. PEG-based solid electrolytes incorporating MOFs are known,^26^–^29^ but they all incorporate lower loadings (<40%) of MOF and their membrane-forming characteristics are rarely discussed in detail. Therefore, UiO-66/PEG MMMs with high MOF content (both 70 and 80 wt% MOF) are reported here to compare with the computational study and to previously reported MMMs. Modeling predicts that the UiO-66/PEG composite possesses excellent compatibility, with significant penetration of the polymer in the first layers of the UiO-66 surface and strong MOF/polymer interactions. This scenario is supported by experimental studies on UiO-66/PEG MMMs that confirm good compatibility, but also find a significant drop of MOF surface area, which may be attributable to surface pore blockage. Together, the computational and experimental studies have been used to identify key parameters that correlate with high MOF/polymer compatibility and establish some general rules to identify MOF/polymer pairs that will likely result in a stable composite material.

## Computational methods

**Fig. 1:**
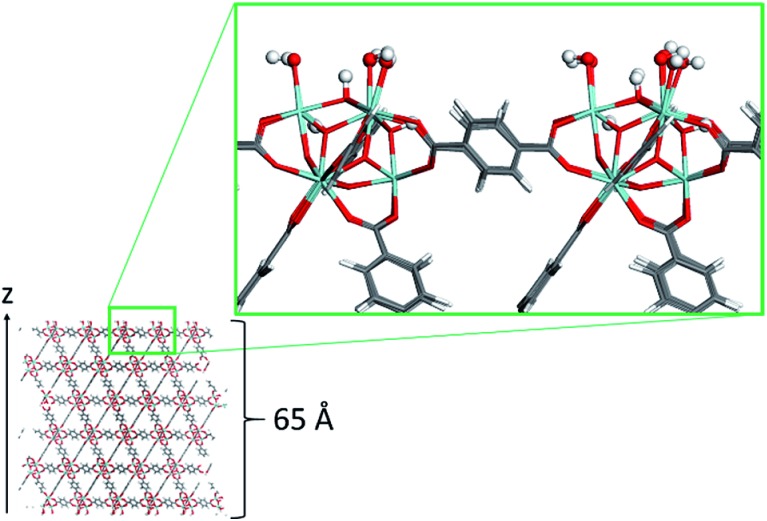
Snapshot of the UiO-66 surface model. Color code: O (red), C (grey), H (white), and Zr (light blue). H and OH surface terminations are highlighted in ball-and-stick representation.

The surface model of the dehydrated UiO-66 material was constructed using the following methodology. The primitive cell was first geometry optimized at the DFT level, using the Quickstep module of the CP2K software.^30^ In these simulations both the positions of the atoms of the framework and the cell parameters were fully relaxed. The PBE functional^31^ was used along with a combined Gaussian basis set and plane wave pseudopotential strategy as implemented in CP2K. A triple zeta Gaussian-type basis set (TZVP-MOLOPT basis set provided with the code)^32^ was considered for all atoms, except for the metal centers, where double zeta functions were employed (DZVP-MOLOPT).^30^ The pseudopotentials used for all of the atoms were those derived by Goedecker, Teter, and Hutter.^33^ These calculations included the semi-empirical dispersion corrections as implemented in the DFT-D3 method, derived by Grimme.^34^ From the resulting optimized structure, sets of Miller indices that would result in a favorable surface cut, were identified *via* the Bravais–Friedel–Donnay–Harker (BFDH) method,^35^–^37^under the assumption that the most stable surfaces are those where the fewer bonds are severed, which led us to select the {101} surface. The surface model slab has a *z*-length of approximately 65 Å (Fig. 1) to avoid the two external surfaces to interact due to periodic boundary conditions. Its dimension along the *x* and *y* axes was 14.45 Å. This surface was reconstructed to ensure dipole neutrality along the *z* axis, and capping the under-coordinated sites resulting from the surface cleavage. The termination scheme considered involves the dissociative adsorption of water (the solvent in the experimental system): Zr atoms are capped by OH^–^ groups, and the remaining H^+^ form a μ_3_-OH group with a framework oxygen atom at the surface (Fig. 1). This surface termination scheme represents one of a number of possibilities where surface interactions with water are concerned, some of which were tested by Planas *et al.*^38^ on a similar Zr-based NU-1000 material.

The final surface model was then geometry-optimized using the same level of theory and parameters as for the optimization of the bulk model. The charges for the material were those published by Yang *et al.*,^39^ except for those of the surface atoms, which were recalculated by the CHELPG method^40^ using a representative DFT-optimized cluster model. In this case, the Gaussian 09 code^41^ was used and the PBE functional^31^ was considered again. The Los Alamos LANL2DZ basis set^42^–^44^ was used to describe the Zr atoms, while the rest of the system was modeled using the 6-31G(d,p) basis set.^45^,^46^ The cluster was constructed using a surface formed by a Zr_6_ unit with the –OH coordinating the Zr atoms and the μ_3_-OH groups, and nine terephthalate ligands attached to the inorganic unit (Fig. S1, Table S1†). A final surface slab model was then constructed for the force field simulations by replicating the surface model in the *x* and *y* directions giving final *x* and *y* lengths of 43.35 Å. This MOF surface model was treated as flexible using the force field parameters described previously by Yang *et al.*^39^

**Fig. 2:**
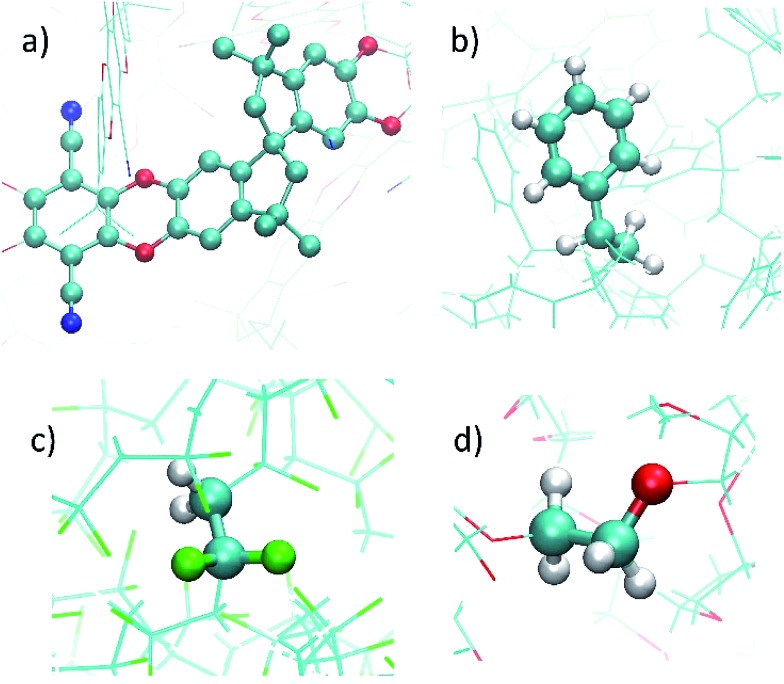
Illustrations of the atomistic models for the different polymers considered in this study: (a) PIM-1, (b) PS, (c) PVDF, and (d) PEG. A monomer is highlighted in ball-and-stick in each image. Color code: O (red), C (cyan), H (white), N (blue), F (green).

The atomistic models for the polymers PIM-1, PVDF, PS, and PEG were constructed using the codes Polymatic^47^ and lammps.^48^ Bonded contributions to the force field were modeled as harmonic potentials for the stretching and bending modes and cosine-based functions for the dihedral angles. Non-bonded interactions were treated as a summation of 12-6 Lennard–Jones (LJ) and Coulomb potentials. The corresponding potential parameters for PVDF and PEG were taken from the DREIDING force field.^49^ For PS and PIM-1, the GAFF force field was used to model bonded interactions^50^ and LJ parameters were taken from TraPPE.^51^ United atom model was considered for PIM-1, and the related potential parameters are those reported in previous studies.^19^ Non-bonded parameters are provided in Tables S2–S5,† and schemes with the atom types of the monomers are presented in Fig. S2–S5.† Table S6† shows the size of the models, all of them are similar in terms of total volume occupied by the polymer, and large enough to avoid the interaction of the two external surfaces of the MOF slab when they are combined. The crossed LJ interactions were computed by using the Lorentz–Berthelot mixing rules in all cases.^52^ The cutoff was set to 12 Å. Coulomb contributions were computed by the Ewald summation, considering ESP partial charges computed by DFT calculations on the monomer. Transferability of the charges from the monomer to the polymer was further tested. The polymer structures are illustrated in Fig. 2. The polymer models were terminated mimicking the experimental monomers, as detailed in the ESI.†

The UiO-66 surface was further combined with the polymers in order to build the different interfaces by applying our previously developed methodology.^19^ The modeled MOF and polymer species were first equilibrated and then the simulations boxes were brought together. Seven cycles of three molecular dynamics (MD) simulations were performed, each of them consisted of two simulations in the NVT ensemble and one in the N*P*_n_*T* ensemble, where *P*_n_ is the pressure component in the direction perpendicular to the slab.

The first simulation in each cycle was conducted at *T*_max_ = 600 K, while for the others it was set to 300 K. Pressure was increased in the first three cycles until reaching a maximum value (*P*_max_) and then decreased up to ambient pressure in the remaining four cycles. Both *T*_max_ and *P*_max_ were selected by applying a similar procedure to the equilibration of the bulk polymers and checking that the correct density was obtained. *P*_max_ was of 50 kbar for PIM-1 and 1 kbar for the others. The higher pressure necessary for achieving a good equilibration of PIM-1 was also previously noted elsewhere.^53^ After these equilibrating cycles, data were collected from ten statistically independent simulations, each one lasting 10 ns, with a time step of 1 fs. The equilibration, interface generation, and production runs procedures were the same as previously applied to the ZIF-8/PIM-1 and ZIF-8/PIM-EA-TB systems.^19^,^20^ Berendsen thermostat and a modified version of the Berendsen barostat that allows for N*P*_n_*T* simulations were used, with relaxation times of 0.1 and 0.5 ps respectively.^54^ For the interface generation and production simulations a modified version of DLPOLY classic was used.^55^

## Experimental methods

### General information

All solvents, starting materials, and polymers were purchased from chemical suppliers and used without further purification (Sigma Aldrich, Arkema, Alfa Aesar, EMD, and TCI). UiO-66(Zr)^22^ was synthesized according to previously-reported methods,^23^ yielding 200 nm truncated octahedral particles (Fig. S17†) with the expected powder X-ray diffraction pattern (Fig. S18†) and a BET area of 1380 ± 60 m^2^ g^–1^ (Table S7, Fig. S19†).^56^

### MMM fabrication

MOF-based MMMs with PEG (*M*_z_ = 900 000 g mol^–1^, PDI = 1.2, purchased from Sigma Aldrich) were fabricated according to a modified method.^21^,^23^ The MOF component was dispersed in acetone (3.5 wt% MOF) *via* ultrasonication for 30 min. The polymer component was dissolved separately in water (3.5 wt%) to a honey-like viscosity. The two component solutions were then combined in the appropriate ratios to yield mixtures containing up to 70 or 80 wt% MOF, which were ultrasonicated for 1 h. The resulting solution was then concentrated by rotary evaporation, removing the acetone, and yielding a homogeneous ‘ink’ of MOF and polymer. The ink was then cast *via* doctor blade onto aluminum foil using an automatic film coater set at a blade height of 800 μm and dried in a 70 °C oven. The membranes were then peeled from the aluminum foil backing using tweezers.

### MMM mechanical analysis

The mechanical integrity of MMMs was assessed in both qualitative and quantitative ways. MMMs that do not form continuous films at 70 wt% MOF (PS, PIM-1) could not be assessed for mechanical properties. MMMs that maintain physical integrity at 70 wt% MOF during drying and could be delaminated as a single piece (PVDF, PEG) were subjected to ASTM standard tensile testing. Tensile strength measurements were conducted per previous reports according to ASTM Standard D882-02 using an Instron® Universal Testing Machine (5965 Dual Column Testing systems, Instron) with a 5 kN load cell in extension mode. Tensile measurements were acquired at an extension rate of 0.005 mm s^–1^ with a sampling rate of 500 ms to generate stress–strain curves, then ultimate tensile strength (UTS) and Young's modulus were further calculated. Tensile data were collected for 5–6 independent samples each and averaged. Sample thicknesses were measured using a Mitutoyo Digital Micrometer (0–25 mm range, 0.001 mm resolution, IP 54 standard) and averaged from 5 independent measurements for each sample.

### N_2_ sorption analysis

Approximately 50 mg of sample (powders of MOF or gently-rolled sections of MMMs) were placed in a tared sample tube and degassed at 105 °C on a Micromeritics ASAP 2020 Adsorption Analyzer until the outgas rate was <5 mm Hg (12–48 h). Post-degas, the sample tube was weighed, and then N_2_ sorption isotherm data was collected at 77 K using a volumetric technique. BET analysis details can be found in the ESI.†

## Results and discussion

**Fig. 3:**
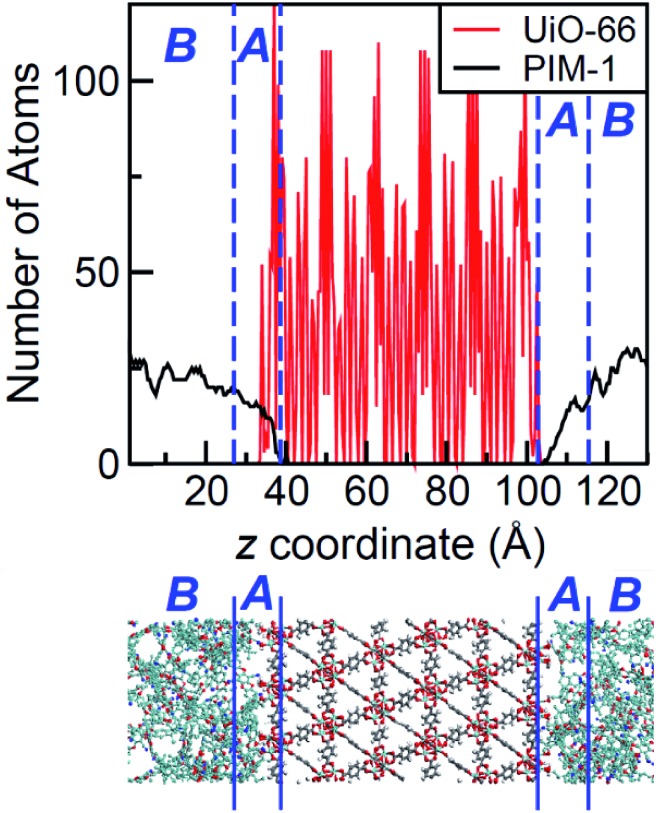
Top: Atomic density profile of PIM-1 (black) and UiO-66 (red) in the direction perpendicular to the surface slab. PIM-1 density fluctuates around a constant value in region B, and drops linearly in region A. Bottom: Schematic of the interface between PIM-1 and UiO-66, aligned with the density profile plot.

**Fig. 4:**
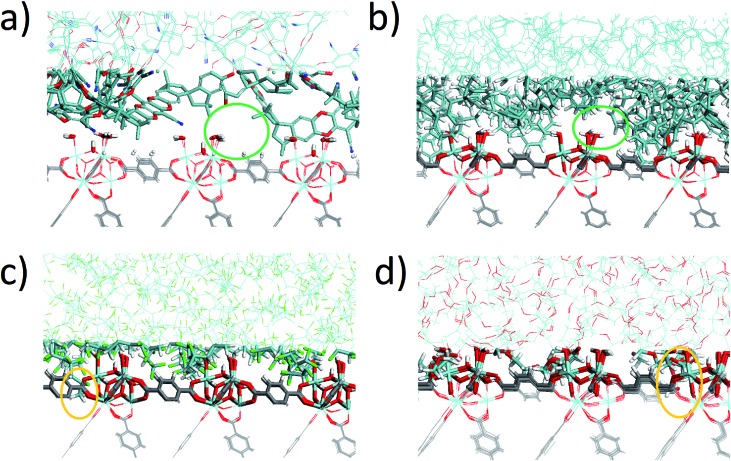
Snapshots of the interfaces for: (a) UiO-66/PIM-1, (b) UiO-66/PS, (c) UiO-66/PVDF, and (d) UiO-66/PEG composites. Colors are opaque in region A, and faded for the rest of the interface. Orange circles mark where polymer end groups penetrate into the open pores of the MOF, and green circles mark the interfacial microvoids.

The density profile as a function of the direction normal to the MOF surface, namely the *z* coordinate, for the UiO-66/PIM-1 interface is shown in Fig. 3. The UiO-66 surface is located at the center of the simulation box, with the polymer phase above and below, as shown in the scheme under the graph. In the proximity of the MOF surface, the polymer density (black line, Fig. 3) decays to zero. At both ends of the box, it fluctuates around a mean-value. In what follows, we will refer to these different polymer regions as A and B respectively. This overall two-region behavior was also observed for the ZIF-8/PIM-1 interface.^19^ The lower limit of region A is taken as the *z* value for the first non-zero polymer density and the upper limit, as that for which the polymer density starts to oscillate. The extension of region A is the distance between these two points in the *z* axis, this will be referred from here onwards as the *z* length A, which is of 15 ± 3 Å for UiO-66/PIM-1. There is a zone within region A where the polymer and the MOF overlap due to the penetration of the polymer into the “pockets” formed by the atomic roughness of the surface. We have identified interfacial microvoids both in region A and B, with equivalent maximum diameters of 13 ± 4 Å and 13 ± 3 Å, respectively, and some degree of interconnection (Fig. 4a, Fig. S6†). We encountered a similar scenario for the UiO-66/PS composite, with the presence of microvoids with diameters of up to 8 ± 1 Å in region A (Fig. 4b, S8 and S9†). In this case, microvoids in region A are larger than those in region B of 6 ± 2 Å. These microvoids are consistent with those seen in our earlier reports^19^,^20^ for ZIF-8/PIM-1 and suggest a similarly non-ideal MOF/polymer interface for UiO-66/PS and UiO-66/PIM-1. When tested experimentally by preparing UiO-66/PS MMMs with a high UiO-66 content (70 wt%), PS-based membranes form cracks during drying and cannot be removed from the substrate without significant disintegration.^23^ This result is commonly noted in the MMM literature, where upper limits of MOF loading are cited based on where MMMs begin to fail physically.^18^,^24^,^57^,^58^ Our modeling results for PS and PIM-1 predict poor interactions between the polymer and filler in these MMMs, with increased rigidity, decreased density, and appearance of microvoids at the MOF/polymer interface. This suggests that the polymer is unable to fully conform to the surface of the filler in these combinations. Hence, when rigid polymers are employed in these MMMs with high MOF loadings, both computational and experimental data indicate poor compatibility of the MMMs.

**Fig. 5:**
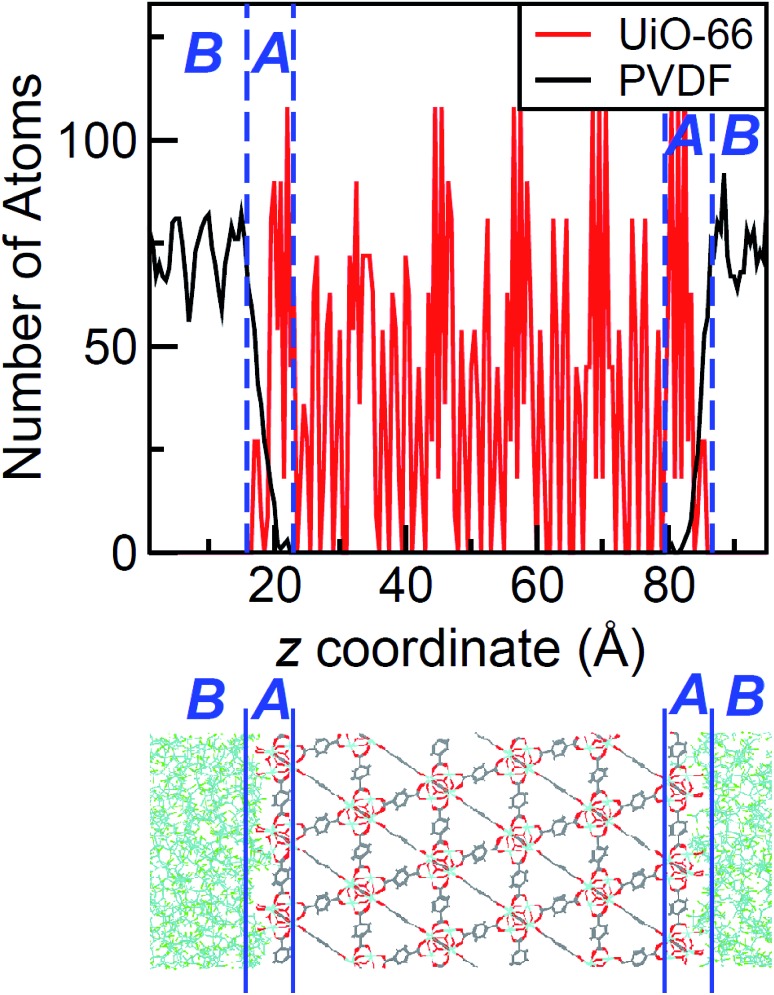
Top: Atomic density of PVDF (black) and UiO-66 (red) in the direction perpendicular to the surface slab. Note the MOF/polymer overlap in region A. Bottom: Schematic of the interface between PVDF and UiO-66, aligned with the density profile plot.

Next, the surface coverage and interfacial porosity of the UiO-66/PVDF interface was modeled. The corresponding density profile depicted in Fig. 5 shows a notable difference compared to that for UiO-66/PIM-1 (Fig. 3). For UiO-66/PVDF, MOF and polymer coexist throughout all region A and there are no interfacial microvoids. PVDF conforms to the morphology of the MOF surface, filling the pockets of surface roughness. Moreover, the polymer terminations penetrate the surface pores of UiO-66. This is illustrated in the snapshots showing a representation of the corresponding interface (Fig. 4c), which is notably different when compared with those obtained for the other MMMs. This microscopic scenario provides an explanation for the excellent MOF/polymer compatibility that was experimentally found for this composite.^21^ Interestingly, the density fluctuations in region B have a larger amplitude for the PVDF-based composite than for PIM-1-based one, suggesting a longer-scale effect, and a stronger interaction with the UiO-66 surface.

The values of *z* length A for all UiO-66/polymer composites studied are listed in Table 1. Those for the PIM-1 and PS-based composites are larger than that for the PVDF-based one. This indicates that it requires a longer distance from the MOF surface for the polymer to start recovering its bulk density.

**Table 1:** Surface coverage parameters for the different UiO-66/polymer interfaces

Polymer	*z* length A (Å)	*ρ*_s_ (Å^–3^)	*λ*_s_
PIM-1	15 ± 3	0.013 ± 0.009	0.2 ± 0.2
PS	9 ± 2	0.05 ± 0.01	0.5 ± 0.1
PVDF	6 ± 1	0.10 ± 0.02	1.0 ± 0.2
PEG	5 ± 1	0.11 ± 0.01	1.0 ± 0.1

Based on this parameter, the affinity of UiO-66 for PVDF (6 ± 1 Å) is higher when compared to PIM-1 (15 ± 3 Å) and PS (9 ± 2 Å), respectively. This comparison holds as well for the ZIF-8/PIM-1 and ZIF-8/PIM-EA-TB interfaces that we explored in previous studies, which have *z* lengths A of 13 ± 1/13 ± 2 Å and 11 ± 1/12 ± 1 Å respectively for interfaces built by considering two different model ZIF-8 surface slabs.^19^,^20^,^59^

In order to quantify the amount of polymer that interacts with the open pores at the MOF surface, we further computed the atomic density of the polymer in the region where it coexists with the MOF, *ρ*_s_, calculated as the number of polymer atoms divided by the volume of the superimposition region. From the comparison of *ρ*_s_ values in Table 1, we conclude that the surface coverage of the UiO-66/PVDF composite is higher than that of UiO-66/PIM-1 and UiO-66/PS MMMs, where microvoids are present. This is another indicator of the improved compatibility of UiO-66/PVDF when compared with PIM-1 or PS.

It could be argued that the higher *ρ*_s_ at the interface for the PVDF-based composite is just a feature originating from PVDF's intrinsically higher density (1.78 g cm^–3^)^60^ when compared to PS (1.04–1.06 g cm^–3^)^60^ and PIM-1 (1–1.2 g cm^–3^).^61^ To assess this, we have computed the normalized density *λ*_s_ which can be calculated by dividing *ρ*_s_ by the bulk polymer density. If the higher density of PVDF in the proximity of the MOF was only due to its intrinsic higher density, *λ*_s_ values would be comparable for the three composites. As shown in Table 1, *λ*_s_ values follow the same trend as *ρ*_s_, indicating that the higher coverage is not due to the higher intrinsic density of PVDF compared to PS and PIM-1, but rather it is related to a real increase in the MOF/polymer affinity. The *λ*_s_ values show that the density of PVDF close to the MOF surface is on average statistically similar to its bulk density, while that for PS is ∼50%, and for PIM-1 only ∼20% of their respective bulk densities.

PIM-1 62 and PS^60^ possess Young's modulus values of 1.26 and 2.25 GPa, respectively, while this value for PVDF is a mere 0.80 GPa. This corresponds to a higher rigidity for PIM-1 and PS when compared to PVDF, which correlates with the presence of interfacial microvoids and decreased surface coverage in their MMMs with UiO-66. This suggests that the flexibility of the polymer might play a crucial role in the MOF/polymer interfacial structure, and thus in their compatibility. Because PEG (Young's modulus of 0.13 GPa) is even more flexible than PVDF, it was predicted that PEG would be a suitable candidate for forming MMMs with UiO-66.

Computations indicate that the UiO-66/PEG interface is similar to that calculated for UiO-66/PVDF: (i) no interfacial microvoids are present, (ii) the polymer adapts its configuration to the morphology of the MOF surface, and (iii) the end groups of the polymer can penetrate into the surface pores of the MOF (Fig. 4d and S13†). From the surface coverage analysis, the UiO-66/PEG composite follows the same tendencies as for UiO-66/PVDF. Specifically, for the UiO-66/PEG the *z* length A is shorter than for most other polymers, and *ρ*_S_ and *λ*_s_ are comparable to those computed for UiO-66/PVDF (Table 1), both of which indicate good surface coverage.

**Fig. 6:**
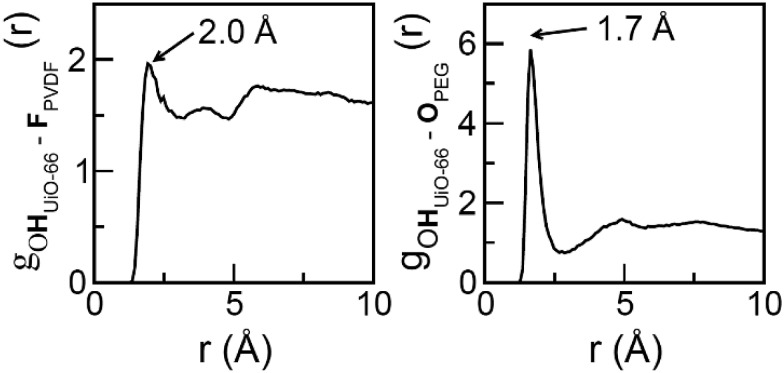
Radial distribution functions for the most preferential MOF/polymer interactions for the UiO-66/PVDF (left) and UiO-66/PEG (right) interfaces.

To further examine the intermolecular interactions in UiO-66/PVDF and UiO-66/PEG, we computed several site-to-site radial distribution functions, *g*_αβ_ (*r*). These functions represent normalized histograms of the distribution of atoms of type β at a given distance of an atom of type α, averaged over all α atoms and the different configurations in a molecular dynamics trajectory. Therefore, peaks are centered at the preferred interaction distances, and the area under the peaks can be related to the probability with which the interaction occurs. Both distance and probability of the interaction are parameters that can be related to its strength. Fig. 6 depicts the main interactions for UiO-66/PVDF and UiO-66/PEG corresponding to the closest MOF/polymer contacts. The most significant interactions involve the hydrogen bonds between fluorine of PVDF and the terminal OH in the MOF surface (F_PVDF_···HO_UiO-66_), and between the oxygen of PEG and the OH groups (O_PEG_···HO_UiO-66_). Fig. 6 shows that the main interaction for the PEG-based composite is characterized by a shorter distance (1.7 Å *versus* 2.0 Å). The integral under the first peak is 3.6 for the PEG case *versus* 2.3 for the PVDF-based composite. This trend supports a stronger MOF/polymer interaction for the UiO-66/PEG system and partially explains why the resulting interface of this composite is so compact.

**Fig. 7:**
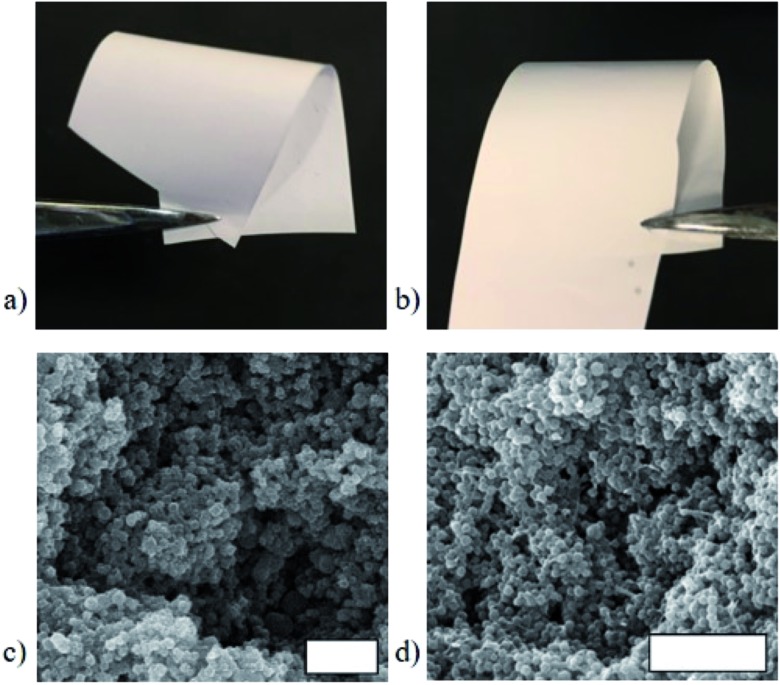
Comparison of the bulk flexibility of 70 wt% MOF MMMs using PEG (a) and PVDF (b) showing very little difference. Similarly, the SEM microstructure of the PEG (c) and PVDF-based (d) MMMs are very similar. Scale bars are 2 μm.

To test the computational prediction, the corresponding UiO-66/PEG MMMs were fabricated at 70 wt% MOF using a previously established method (see ESI†).^21^,^23^ UiO-66/PEG MMMs are flexible and visibly continuous at 70 wt% UiO-66, and they are similar in appearance to UiO-66/PVDF MMMs at the same MOF loading (Fig. 7, S20 and S21†). Powder X-ray diffraction (PXRD) spectra of PEG MMMs show retention of the crystallinity of the MOF in the MMM (Fig. S22†). In SEM cross-section images, PEG and PVDF MMMs are essentially indistinguishable, with both showing the MOF-dominant, loosely-packed morphology seen in previous studies (Fig. 7 and S23–S25†).

**Fig. 8:**
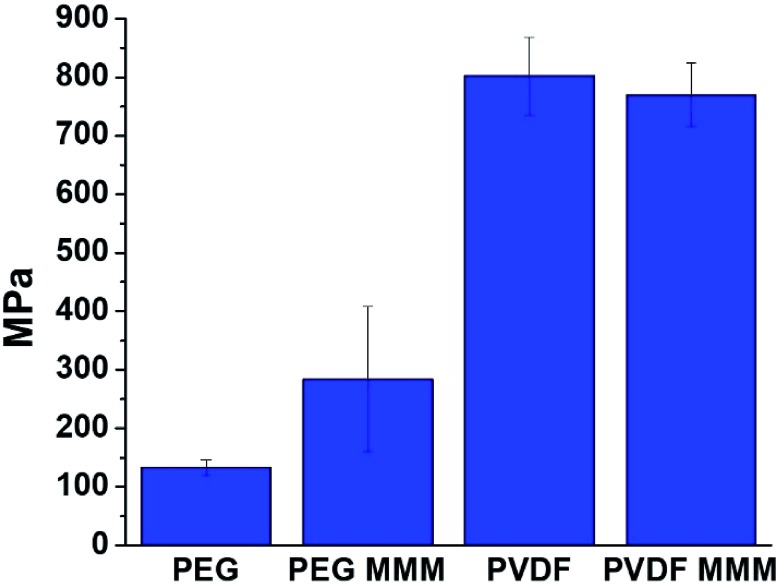
Young's modulus values for PVDF, PEG, and their respective 70 wt% UiO-66 MMMs. The increase in Young's modulus for the PEG-based MMM (UiO-66/PEG) when compared to pure PEG films indicates favorable MOF/polymer interactions.

Although PVDF and PEG-based MMMs appear similarly stable when handled and their interfaces show similar features in our computational studies, stress–strain curves of PVDF and PEG systems show that pure PVDF membranes are significantly stronger and more rigid than pure PEG membranes (Fig. 8, Table S8, Fig. S26†). Significant differences between the mechanical properties of MMMs from PVDF and PEG with 70 wt% UiO-66 are also found, both in strength (UTS) and rigidity (Young's modulus).

Because the initial mechanical properties of the pure polymer membranes vary greatly, it is difficult to extract meaningful information about MOF/polymer interactions by directly comparing PVDF- and PEG-based MMMs mechanical properties. However, previous work has shown that we can gain insight into the interactions between filler and matrix by comparing a pure polymer with its composite form.^58^,^63^–^67^ By comparing mechanical properties between the pure polymer membranes and the corresponding MMMs, we can hypothesize about the differences between PVDF and PEG MMMs, while bearing in mind that differences in the inherent strength and rigidity of these polymers also play a role.

In both PVDF and PEG systems, the UiO-66 based- MMMs have much lower UTS values than that of the corresponding pure polymer film. In many composite materials, the addition of fillers increases the UTS of the composite, if filler loading is low.^68^–^70^ However, as filler content is increased, filler aggregates can act as ‘stress concentrators’^70^ and lead to a reduction in UTS,^21^,^23^,^64^,^70^ which is the result in both the PVDF and PEG MMMs reported here (Table S8, Fig. S26†). Despite UTS decreases in both systems, MMMs of both PEG and PVDF containing 70 wt% UiO-66 maintain sufficient stability to be handled, rolled, and twisted.

We begin to see differences in the mechanical behavior between PVDF and PEG MMMs when Young's modulus is examined. At low MOF loadings (<20–30 wt% MOF), prior studies on MOF-based MMMs report that Young's modulus values tend to increase sharply when compared to the base polymer,^21^,^23^,^58^,^64^–^67^ indicating that MMMs are more rigid than their pure polymer counterparts. This increased rigidity is attributed to strong MOF/polymer interactions.^58^,^64^–^67^ A comparison of the Young's modulus of a pure PEG film with that of its corresponding 70 wt% UiO-66 MMM shows that the MMM is significantly more rigid than the starting polymer, with Young's modulus values increasing from 133 ± 13 MPa in pure PEG to 284 ± 124 MPa for the 70 wt% MOF MMM (Fig. 8, Table S8†). When PVDF is compared with its respective MMM, the rigidity of the pure polymer is almost identical to that of the MMM, showing only a slight decrease of 32 MPa from 802 ± 66 MPa for PVDF to 770 ± 54 MPa for the MMM (Fig. 8, Table S8†). The differences in Young's modulus trends suggest that the reduced rigidity of PEG combined with computationally-predicted strong UiO-66/PEG interactions may explain the increase in rigidity seen in the PEG-based MMM.

Thermal gravimetric analysis (TGA) data of PEG and PVDF-based MMMs differ from the respective base polymers, suggesting incorporation of MOF into these polymers alters the properties of the polymer (Fig. S27 and S28†). As the amount of MOF in the PEG MMMs increases from 0% incorporation (a PEG-only membrane) to 30, 50, 70, and 80 wt% UiO-66, the degradation temperature (*T*_d_) of PEG decreases from 388 °C for pure (0 wt% MOF) PEG to 324 °C for an 80 wt% MOF MMM, a total decrease of 64 °C (Fig. S27†). A slightly different trend is observed for PVDF MMMs. A 60 °C drop in *T*_d_ is observed as MOF is included, when comparing a pure, 0 wt% MOF PVDF membrane to 30 wt% UiO-66 in PVDF. As the MOF amount is increased to 50 wt%, a *T*_d_ increase of 20 °C is observed, and a further increase of 10 °C occurs in the 70 wt% MOF/PVDF MMM (Fig. S28†). The initial drop in stability upon addition of 0–30 wt% MOF is also seen in a related study on zeolite incorporation into PVDF.^71^ In both PEG and PVDF MMMs, the overall decrease in the thermal stability of the films compared to polymer-only films can be attributed to interruptions in long-range polymer interactions by the MOF additive.

A comparison of DSC traces reveals small differences between PVDF-based MMMs and PEG-based MMMs (Fig. S29 and S30†). A 2 °C increase in melting temperature (*T*_m_) from pure PEG to 30 wt% MOF in PEG is observed, followed by decreases in *T*_m_ as more MOF is added (Fig. S29†). In the PVDF system, *T*_m_ decreases as MOF is incorporated up to 70 wt% (Fig. S30†). The overall decreases in *T*_m_ in both systems again likely correlates with reduced polymer–polymer interactions as the MOF component is increased. The slight increase observed in the *T*_m_ of 30 wt% MOF/PEG MMM could be a sign of increased stability of this MMM over the base polymer, perhaps due to MOF-polymer interactions, although the small magnitude of this change make conclusions from this data speculative at best.

Additional differences in the UiO-66/PVDF and UiO-66/PEG MMMs are seen when comparing the accessible internal surface area of the MMMs by N_2_ sorption. While the 70 wt% UiO-66/PVDF-based MMM retains the internal surface area of the MOF component, its PEG-based counterpart shows no accessible internal surface area at 77 K. This observation is consistent with the strong UiO-66/PEG interactions seen computationally (Fig. 5 and 6) and mechanically (Fig. 8), and suggests that the surface pores of the MOFs may be blocked by some combination of high surface contact (and concomitant pore penetration by polymer) and strong MOF/polymer interactions. UiO-66/PEG MMMs fabricated with 80 wt% MOF begin to recover some of the surface area of the MOF, but only ∼50% the expected capacity based on the amount of MOF present (Table S7, Fig. S31†).

## Conclusions

The microscopic structure of UiO-66/polymer interfaces was systematically investigated by computational methods combining force field and quantum-based molecular simulations. Our results suggest that the rigidity of the polymer has a negative impact in the MOF/polymer compatibility. These findings are consistent with what has been proposed by Koros *et al.* for different nanoparticle/polymer composites.^72^–^74^ Highly compatible systems are found with polymers that show Young's modulus values less than 1 GPa. Polymers that meet this criterion can readily adapt to the morphology of the MOF surface, filling the pockets formed by the atomic roughness of the surface, with chain ends penetrating into the surface MOF pores. This computationally predicted microscopic scenario was evidenced by UiO-66/PVDF and UiO-66/PEG composites, which yield flexible, continuous MMMs with sufficient tensile strength and rigidity to yield useful films at 70 wt% MOF. In contrast, poorly compatible systems such as UiO-66/PS and UiO-66/PIM-1 exhibit interfacial microvoids, which increase the interaction distance between the MOF and the polymers. Consistent with these calculations, UiO-66/PS and UiO-66/PIM-1 generate brittle, fragile MMMs^18^,^23^ that prevented tensile strength testing and verification.

MOF/polymer compatibility can be also tuned by chemical functionality of the two components. Simulations suggest a plausible microscopic explanation for the difference between UiO-66/PVDF and UiO-66/PEG MMMs based on the difference in the strength of these interactions. Both polymers penetrate the open pores of UiO-66. However, for UiO-66/PEG, hydrogen bonding between the MOF and the polymer is stronger, making the interface more compact, and resulting in blockage of the MOF pores, while UiO-66/PVDF makes strong interactions, but without pore blockage. Mechanical testing brings to light further differences between UiO-66/PVDF and UiO-66/PEG MMMs, with UiO-66/PVDF showing no change in rigidity as large amounts of MOF are incorporated, while the rigidity of PEG membranes more than doubles upon incorporation of MOF, providing more evidence of a strong UiO-66/PEG interface. These results suggest that from the point of view of applications, a balance between compatibility and performance is needed: the MOF/polymer pair should be compatible enough to give robust membranes with high MOF loadings, but avoid interactions that lead to pore blocking.

## Conflicts of interest

There are no conflicts to declare.

## Supplementary Material

Supplementary information
