# Supplementary material for: Understanding the origins of metal–organic framework/polymer compatibility
Source: Chem Sci. 2017 Oct 27;9(2):315–24. doi: 10.1039/c7sc04152g (PMC5868319; doi:10.1039/c7sc04152g)
Supplement: Supplementary file 1 [file SC-009-C7SC04152G-s001.pdf]

Electronic Supplementary Material (ESI) for Chemical Science.
This journal is © The Royal Society of Chemistry 2017

Supporting Information

Understanding the Origins of Metal-Organic Framework/Polymer Compatibility

Rocio Semino[†], Jessica C. Moreton[‡], Naseem A. Ramsahye[†§], Seth M. Cohen[‡*], Guillaume Maurin[†*]

† Institut Charles Gerhardt Montpellier UMR 5253 CNRS, Université de Montpellier, Place E. Bataillon, 34095 Montpellier Cedex 05, France.

‡ Department of Chemistry and Biochemistry, University of California, San Diego, La Jolla, California 92093-0358, United States of America.

§ Institut Charles Gerhardt Montpellier, UMR 5253 CNRS, UM, ENSCM, 8 rue de l'Ecole Normale, 34296 Montpellier Cedex 05, France.

Table of Contents

Computational details…………………………………………………………….. S2
Surface models………………………………………………………………… S2
Polymer models………………………………………………………….......... S3
Additional modeling results………………..……………………………………… S8
UiO-66/PIM-1 interface……………………………………………………….. S8
UiO-66/PS interface..………………………………………………………….. S11
UiO-66/PVDF interface……………………………………………………….. S13
UiO-66/PEG interface..………………………………………………………... S15
Experimental details……………………………………………………………… S20
Synthetic procedures………………………………………………………… S20
Materials characterization…………………………………………………… S20
Additional experimental results………………………………………………….. S22
References………………………………………………………………………... S31

A) COMPUTATIONAL DETAILS

Surface model

In the following, we provide an illustration of the cluster used for computing the surface charges for the UiO-66 model and the list of atomic partial charges along with the non-bonded Lennard-Jones (LJ) potential parameters used for the Molecular Dynamics simulations. A full list of the bonded terms for the flexible UiO-66 force field can be found in the previous study by Yang *et al.*[1]

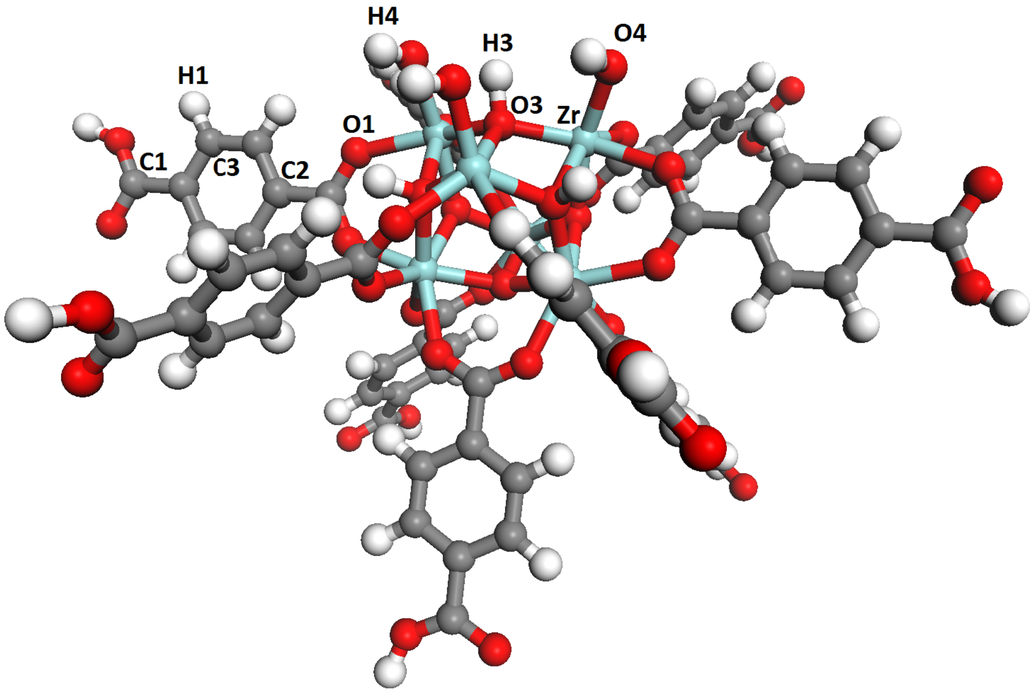

Figure S1. Scheme of the cluster used for the UiO-66 surface charges calculations. Color code: O (red), C (grey), H (white) and Zr (light blue).

Table S1. LJ parameters and charges for the UiO-66 surface model.

Atom type	ε_{ii} *(kcal/mol)*	σ_{ii} *(Å)*	q_i *(e)*
C1	0.0951	3.473	+0.6370
C2	0.0951	3.473	-0.0750
C3	0.0951	3.473	-0.0580
H1	0.0152	2.846	+0.1130
H3	0.0152	2.846	+0.2902
H4	0.0000	2.846	+0.3902
O1	0.0600	3.118	-0.5995
O3	0.0600	3.118	-0.9920
O4	0.0600	3.118	-0.6800
Zr	0.0690	2.783	+1.9581

Polymer models

Figures S2, S3, S4 and S5 show schemes of the monomers of the polymer of intrinsic microporosity 1 (PIM-1), polystyrene (PS), poly(vinylidene fluoride) (PVDF) and polyethylene glycol (PEG) respectively. The corresponding non-bonded parameters are included in Tables S2, S3, S4 and S5.

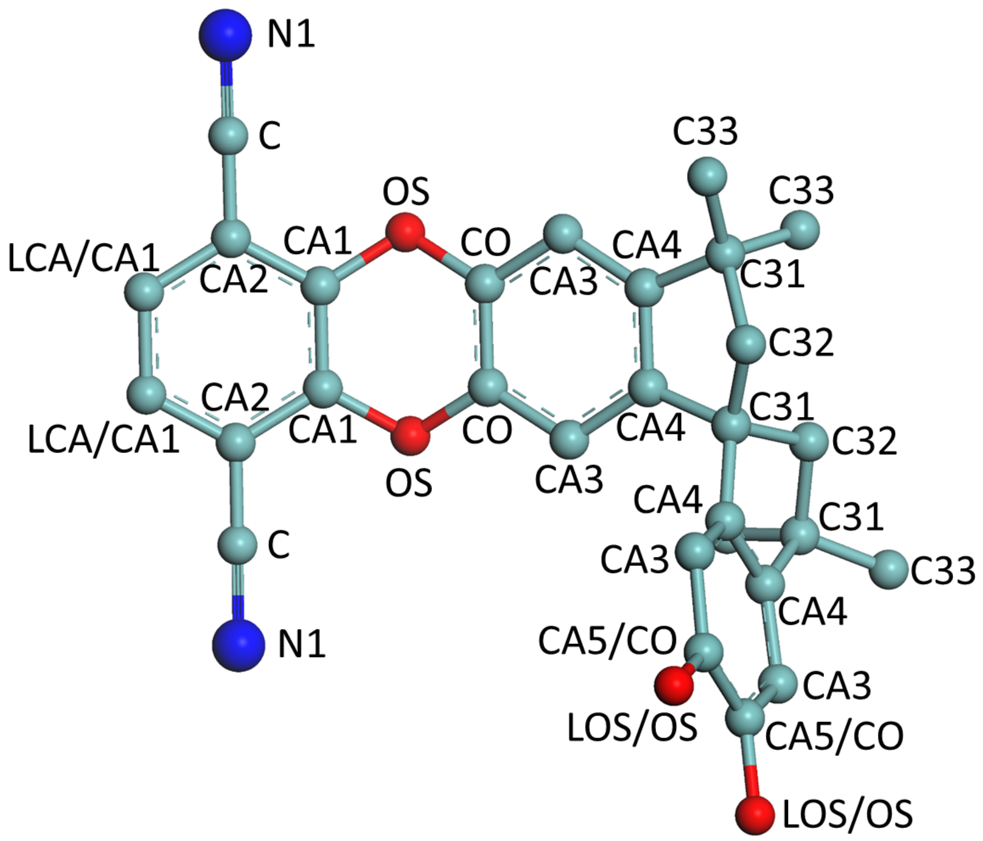

Figure S2. Scheme of the PIM-1 monomer model. Color code: O (red), C (cyan), H (white), N (blue), F (green).

Table S2. Atom types, LJ parameters[2] and charges[3] for the PIM-1 model.

Atom type	ε_{ii} *(kcal/mol)*	σ_{ii} *(Å)*	q_i *(e)*
CA1	0.0636	3.600	+0.085
CA2	0.0435	3.880	-0.287
CA3	0.1050	3.695	-0.144
CA4	0.0435	3.880	-0.044

CA5	0.0636	3.600	+0.187
OS	0.1452	2.600	-0.200
C	0.1243	3.550	+0.427
N1	0.1243	2.950	-0.401
C31	0.00104	6.400	+0.562
C32	0.1059	3.890	-0.280
C33	0.2033	3.750	-0.133
CO	0.0636	3.600	+0.285
LCA	0.0636	3.600	+0.185
LOS	0.2345*	3.040	-0.538
F	0.2286	2.850	-0.100
HOH	0.000	0.000	+0.436

*Note that this parameter was incorrect in the SI of Ref. 3, the correct value is provided here.

Some of the atoms in Figure S2 contain two labels, these atoms change types depending on whether the monomer is a termination of the polymer chain (first label) or whether it is bonded to another monomer (second label). When they are terminations, they are labeled as LCA, LOS, and CA5. This is done in order to have realistic values for the charges, since for example the terminating O (LOS) is part of an OH group, and as such, it is much more electronegative than the ether O in the middle of the chain (OS). LOS are bonded to hydrogen atoms of type HOH, while the terminating carbon LCA are bonded to fluorine atoms of type F. These mimic the experimental terminations of the polymer chains and guarantee electro-neutrality. The PIM-1 model that we used in this work consists of a single chain of 85 monomers, so there are only two terminations. This corresponds to a total of 2 atoms of each type LCA, F, CA5, LOS and HOH.

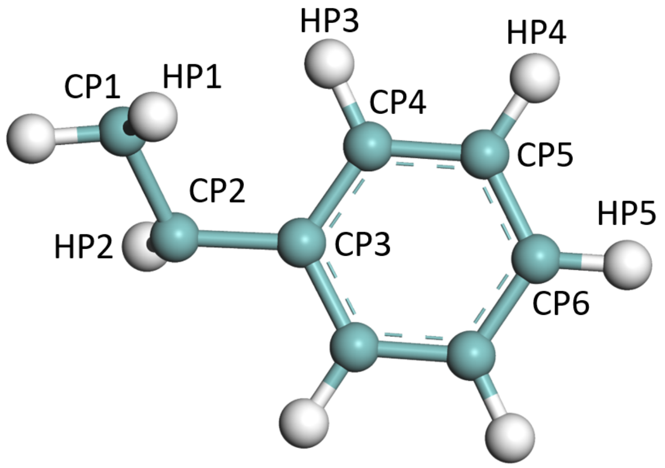

Figure S3. Scheme of the PS monomer model. Color code as in Figure S2.

Table S3. Atom types, LJ parameters[2] and charges for the PS model.

Atom type	ε_{ii} ***(kcal/mol)***	σ_{ii} ***(Å)***	q_i ***(e)***
CP1	0.0951	3.473	-0.360
CP2	0.0951	3.473	-0.050
CP3	0.0951	3.473	+0.183
CP4	0.0951	3.473	-0.230
CP5	0.0951	3.473	-0.070
CP6	0.0951	3.473	-0.200
HP1	0.0152	2.846	+0.124
HP2	0.0152	2.846	+0.059
HP3	0.0152	2.846	+0.144
HP4	0.0152	2.846	+0.138
HP5	0.0152	2.846	+0.156

The PS chains were terminated by adding an H atom at each end as in the experimental monomers. For the CP1 terminations, the H atom added was of type HP1 (charge +0.124 e), and in order to maintain the charge neutrality, the terminal CP1 atom charge was changed from -0.360 e to -0.484 e. The CP2 terminations were capped by an H atom of the type HP2 (charge +0.059 e), and the neutrality was achieved by changing the terminal CP2 atom charge from -0.050 e to -0.109 e.

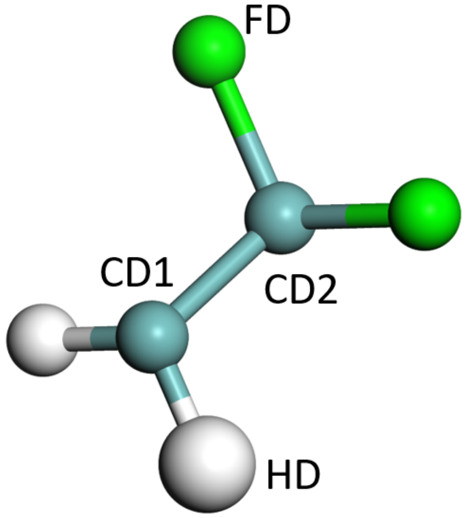

Figure S4. Scheme of the PVDF monomer model. Color code as in Figure S2.

Table S4. Atom types, LJ parameters[4] and charges for the PVDF model.

Atom type	ε_{ii} ***(kcal/mol)***	σ_{ii} ***(Å)***	q_i ***(e)***
CD1	0.0951	3.473	-0.588
CD2	0.0951	3.473	+0.614
HD	0.0152	2.846	+0.208
FD	0.0725	3.093	-0.221

The PVDF chains were terminated by adding an H atom in the terminal CD1 and an F atom in the terminal CD2. The LJ parameters were those for the HD and FD types, but the charges were slightly modified to achieve charge neutrality: a charge of +0.2145 was assigned to the terminal H and one of -0.2145 to the terminal F.

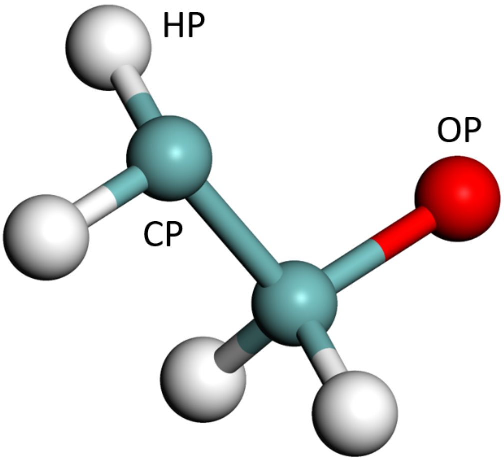

Figure S5. Scheme of the PEG monomer model. Color scheme as in Figure 2.

Table S5. Atom types, LJ parameters[4] and charges for the PEG model.

Atom type	ε_{ii} ***(kcal/mol)***	σ_{ii} ***(Å)***	q_i ***(e)***
CP	0.0951	3.473	+0.060
HP	0.0152	2.846	+0.066
OP	0.0957	3.033	-0.384
HT	0.0001	3.033	+0.192

The PEG chains were terminated by adding an H atom to the terminal OP and an OH group to the terminal CP. LJ parameters and charges for the O in the terminal OH groups were those of OP type, but the terminal H atoms (called HT in Table S5) were assigned different LJ parameters according to DREIDING force field[4] to take into account the possibility of H-bonding, and the charge required to maintain electro-neutrality.

Charges of the polymers were computed by DFT calculations on the monomers using PBE functional[5] and DNP basis set[6] in the *Dmol*^{3} module[7] of Materials Studio.[8] Charges for different atoms of the same force field type were averaged, and slightly modified in order to yield an electro-neutral monomer. In the case of PIM-1, all hydrogens were explicitly included in the calculations, and their charges were then added to the carbons to which they are bonded, to generate the united atoms charges. The transferability of the computed charges was tested in all cases by performing a similar calculation for the corresponding trimer, and it was found to be satisfactory in all cases.

Bonded parameters for PVDF and PEG are those of the DREIDING force field,[4] while those for PS were taken from GAFF.[9] The sizes and polydispersity of all polymer models are shown in Table S6. All models occupy similar volumes, and they are large enough to prevent interaction of the MOF slabs by periodic boundary conditions when put together.

Table S6. Size of the polymer models in terms of chains, total number of monomers and total number of atoms.

Polymer	*Chain details*	*Monomers*	*Atoms*
PIM-1	1 (85 monomers)	85	2979
PS	13 (10 to 56 monomers)	361	5776
PVDF	17 (15 to 164 monomers)	988	5928
PEG	9 (22 to 179 monomers)	991	6937

Since the density of PIM-1 is lower than that for the other polymers, a smaller model (in terms of total number of atoms) is enough to fill a similar volume. Regarding the polydispersity, it has been shown in a previous study on ZIF-8/PIM-1 composites that the obtained interface using a polydisperse PIM-1 model is similar to that obtained using a single chain of similar size.[3]

B) ADDITIONAL MODELING RESULTS

UiO-66/PIM-1 interface

Figure S6 shows number and volume distribution of the pores according to two different methodologies: the *v_connect* methodology[10] and that proposed by Bhattacharya and Gubbins.[11] The former involves probing the voids with a model molecule of defined size, in this case positronium (black) and nitrogen (red), with diameters of 2.2 and 3.64 Å respectively. In this context, what is probed is in fact the free volume, and then a diameter is computed by associating this volume to an isochoric sphere. This means that if two voids are interconnected by a narrow tunnel of the size of one of these probing molecules, they will be considered as one void by this method. The second method gives the pore size distribution obtained by probing the free volume with a probe of increasing diameter. Thus, interconnected voids will be considered as separate. It follows that if the voids are more or less spherical, similar void distributions will be obtained by the two methods providing that they are not interconnected. Otherwise, the voids distribution with the *v_connect* method will

give a wider range (and larger maximum values), and this will be a sign of interconnectivity between the voids.

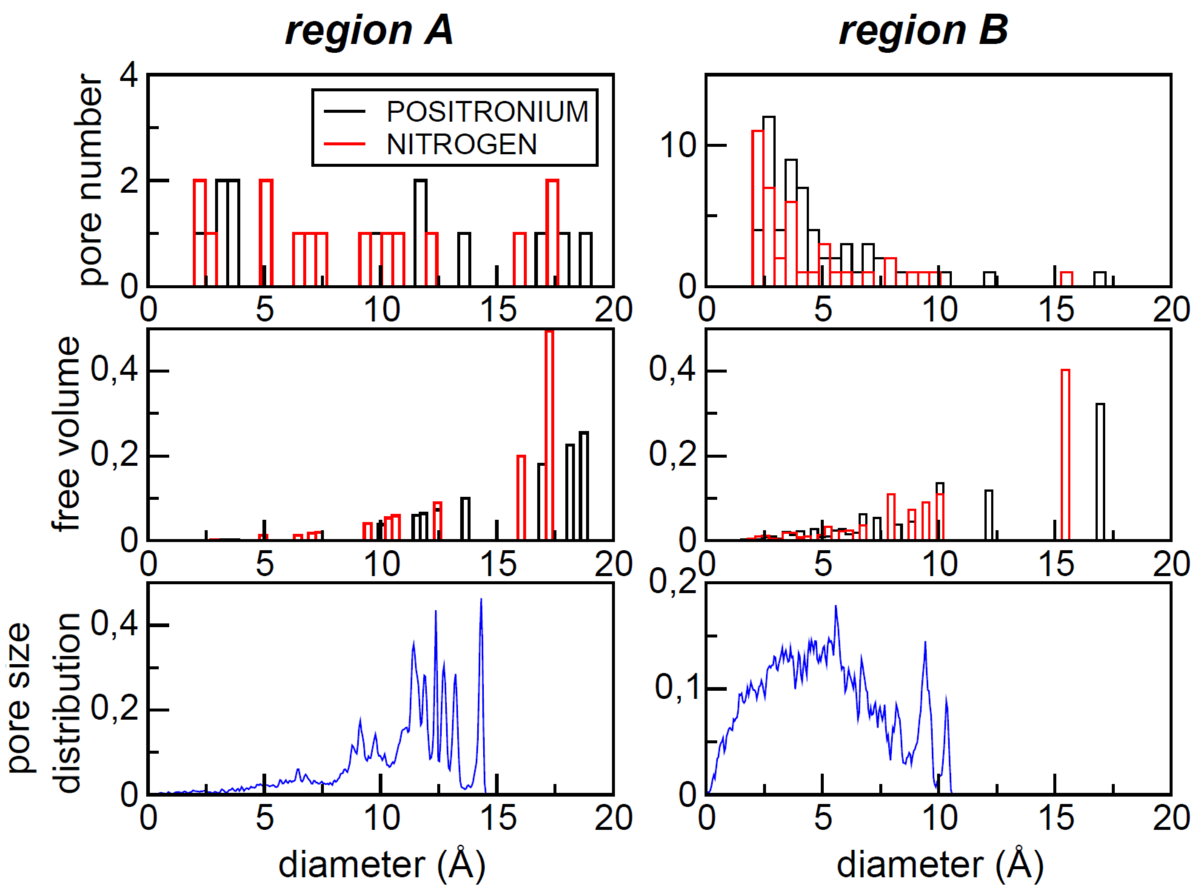

Figure S6. Histograms characterizing the pores in region ***A*** (left) and region ***B*** (right) for a representative configuration of the model UiO-66/PIM-1 interface. Number of pores and free volume fraction (top and middle) as a function of the diameter of the void. Bottom: pore size distribution.

The pore size distribution is plotted in the bottom panels. In the left, we can see that the pore distribution for region ***A*** spans the range of diameters of up to 14 Å. Since we have performed 10 independent runs for each system, and there are two regions ***A*** in each, there are 20 maximum diameters values. If we take into account all of them, the average value of maximum pore diameter is 13 Å, with an error of 3 Å (computed as the standard deviation of all collected values). Region ***B*** also shows microvoids of similar size 13±3 Å. There is interconnectivity between the voids in both regions, as evidenced by the larger diameter values shown in the upper and middle panels, as a result of the *v_connect* analysis (19 and 17 Å respectively in the plots).

We have also analyzed the interactions that hold the PIM-1 and the UiO-66 surface together, these are shown in Figure S7. The main interactions are the hydrogen bonds between the nitrogen of the CN_{PIM-1} group with the OH_{UiO-66} and the less exposed μOH_{UiO-66} groups, with distances of 1.8 and 2.5 Å respectively (see left plots). The intensities of the main peak in the former is high (around 4), indicating a strong interaction. Similar interactions were found with the terminations of ZIF-8 in a previous study.[3] There are also interactions between the ether O_{PIM-1} and the μOH_{UiO-66} groups, with distances varying between 3 and 4 Å.

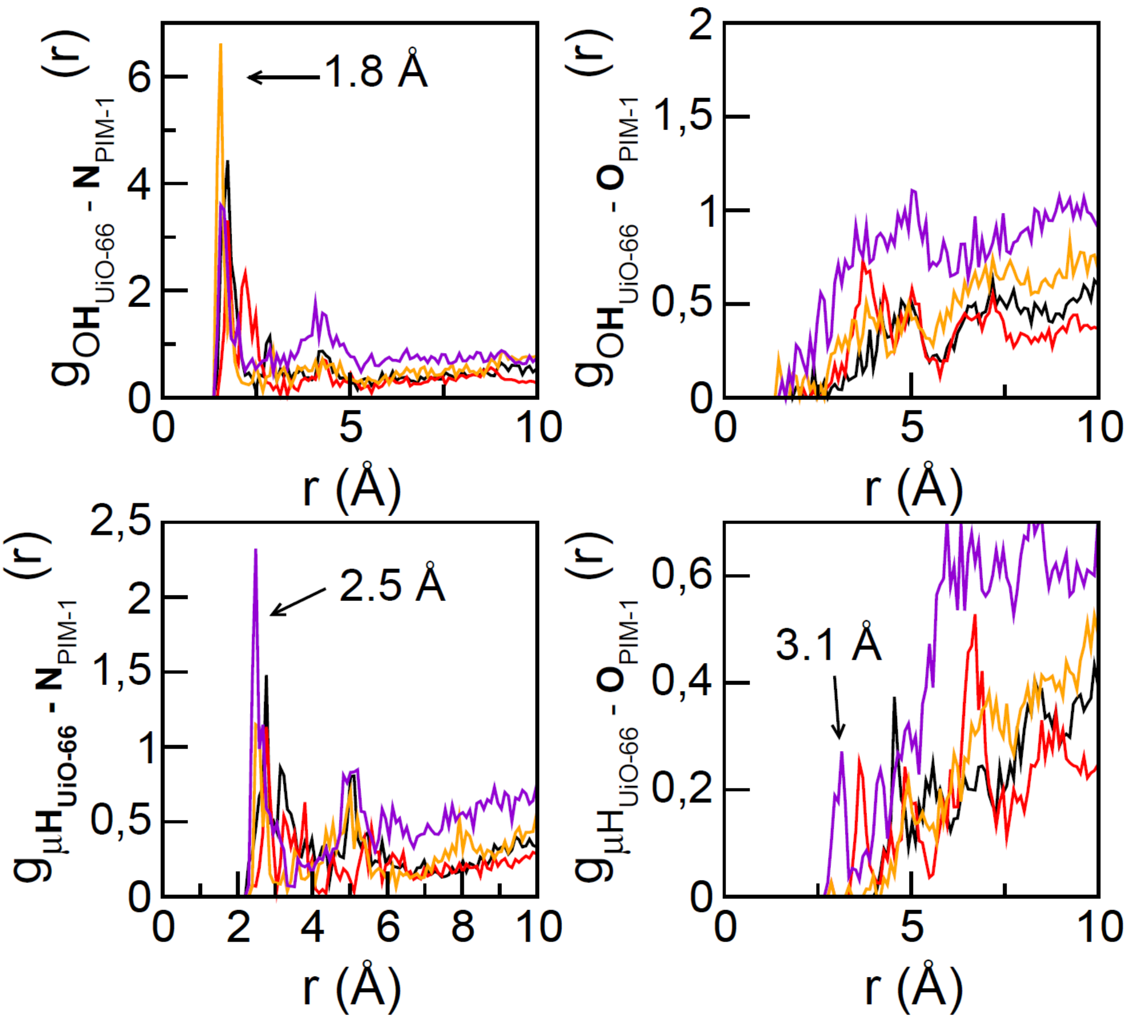

Figure S7. Radial distribution functions between UiO-66 terminations and PIM-1. The different colored curves represent results from different molecular dynamics simulations.

UiO-66/PS interface

The density profile and the distribution and size of the pores in the polymer phase in UiO-66/PS are presented in Figures S8 and S9 respectively.

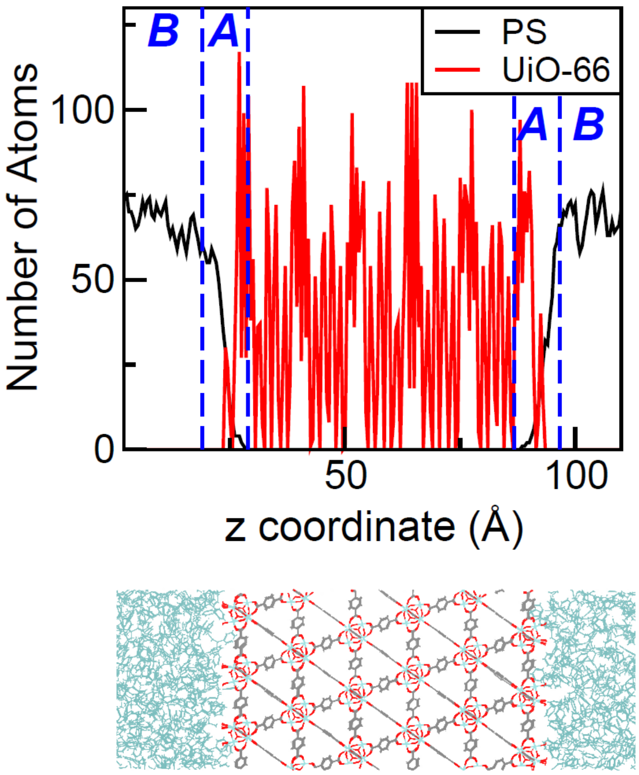

Figure S8. Atomic density of PS (black) and UiO-66 (red) in the direction perpendicular to the surface slab. A scheme for the interface is also provided (color code as in Figures S1 and S3).

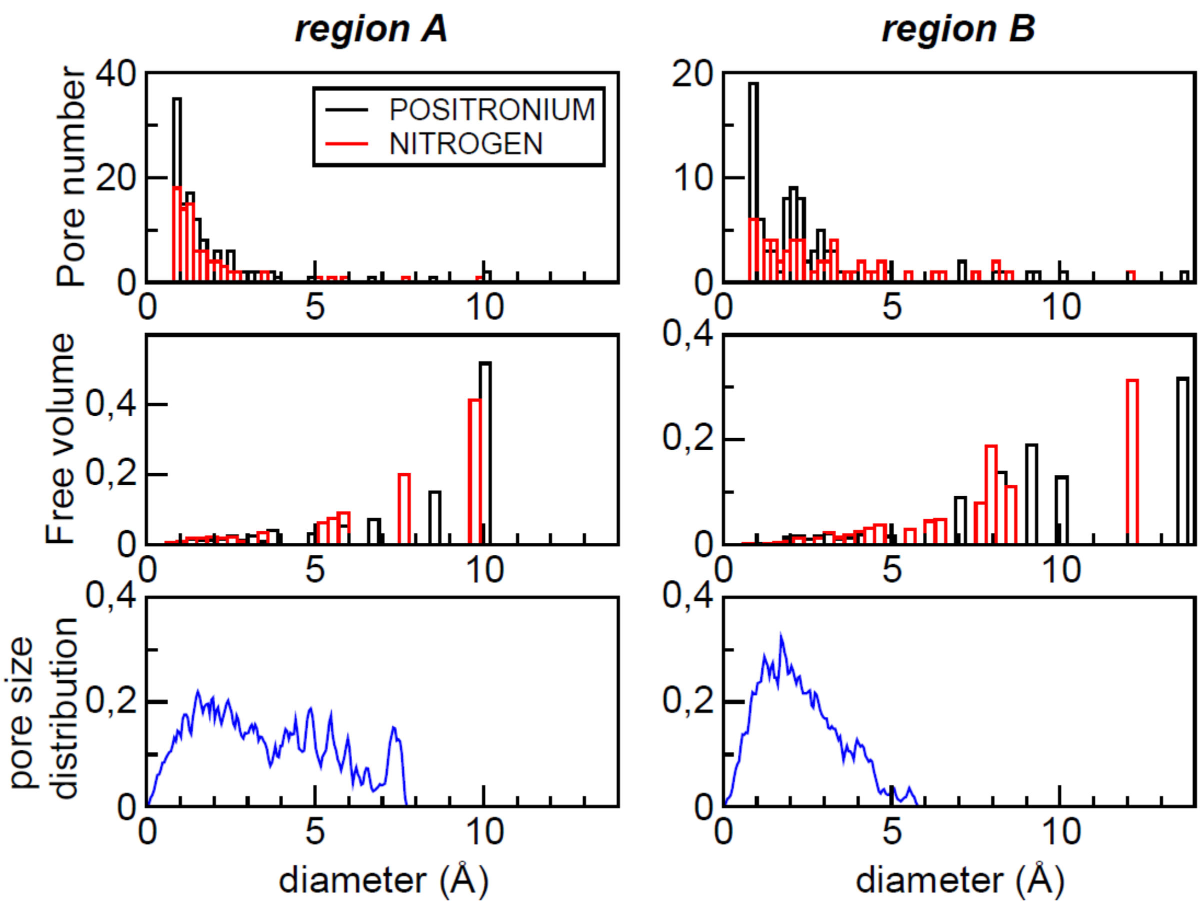

Figure S9. Histograms characterizing the pores in region ***A*** (left) and region ***B*** (right) for a representative configuration of the model UiO-66/PS interface. Number of pores and free volume fraction (top and middle) as a function of the diameter of the void. Bottom: pore size distribution.

The behavior of the density profile is analogue to the UiO-66/PIM-1 case, with the same two-regions and the polymer density dropping before the appearance of the first MOF atoms (part of region ***A*** where MOF and polymer do not superimpose). The limits of ***region A*** are taken to be the *z* values where the polymer has zero density and that from which it starts to oscillate in all cases.

The bottom panels of Figure S9 depict the pore size distribution. Pores in region ***A*** have maximum diameter of 8 Å, while in region ***B*** they are slightly smaller, up to 6 Å. In both regions, there is some degree of connectivity between the voids, as evidenced by the larger diameter values obtained by the *v_connect* methodology (10 and 14 Å for region ***A*** and ***B*** respectively), which are shown in the top and middle panels.

The intermolecular interactions in the composite have been identified by computing site-site radial distribution functions for several MOF/polymer pairs (Figure S10).

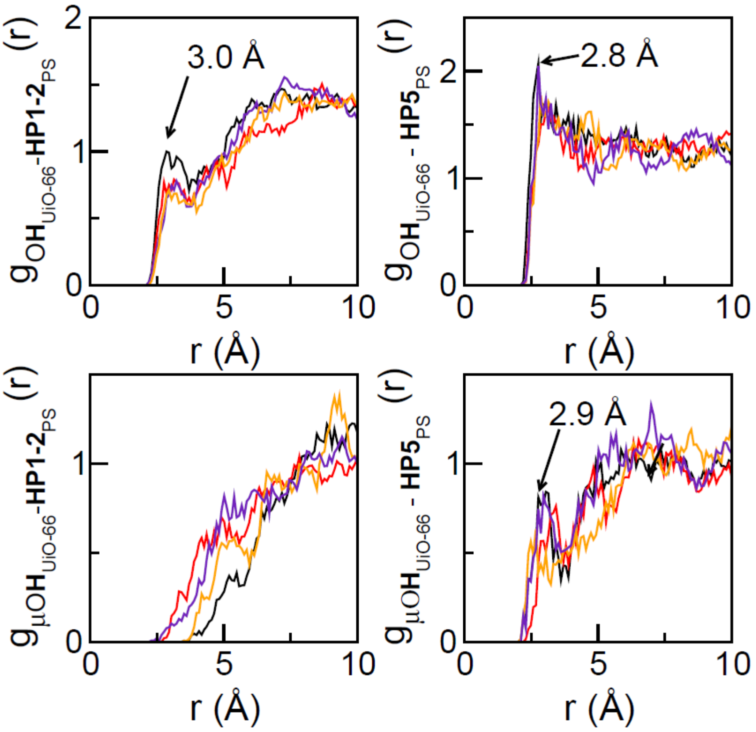

Figure S10. Radial distribution functions between UiO-66 terminations and PS aliphatic and p-aromatic hydrogen. The different colored curves represent results from different molecular dynamics simulations.

Several MOF/polymer interactions can be found. The most external $OH_{UiO\text{-}66}$ groups interact both with the aliphatic hydrogens ($HP1_{PS}$ and $HP2_{PS}$ in Figure S3, 3 Å distance) and with the aromatic ones, particularly with the $HP5_{PS}$ site (distance of 2.8 Å) (see top left and right panels respectively). $HP5_{PS}$ sites also interact with the less exposed $H_{UiO\text{-}66}$ atoms that are bonded to the $\mu O_{UiO\text{-}66}$ and come from the water dissociation considered for the capping (2.9 Å). These latter interactions are what binds the polymer to the "pockets" formed by the morphology of the UiO-66 surface.

UiO-66/PVDF interface

The distribution and sizes of the pores in the polymer phase of UiO-66/PVDF were computed as explained above, results are presented in Figure S11.

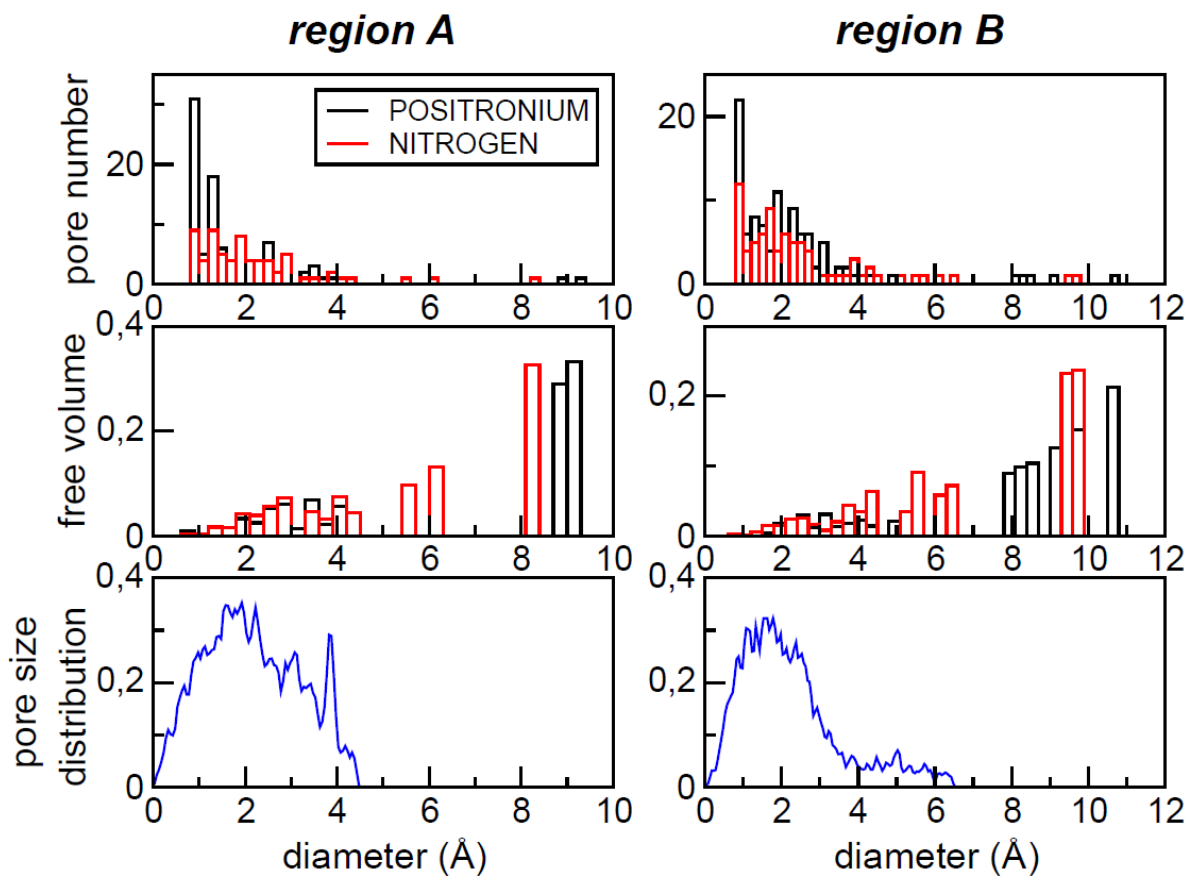

Figure S11. Histograms characterizing the sizes of the voids in region ***A*** (left) and region ***B*** (right) for a representative configuration of the model UiO-66/PVDF interface. Number of pores and free volume fraction (top and middle) as a function of the diameter of the void. Bottom: pore size distribution.

Pores in region ***A*** are of similar size than in region ***B***, with maximum diameter of 4.5 Å. There are some voids that are even larger within region ***B*** (up to 6.5 Å diameter) but they appear with low probability, and their absence in region ***A*** could be due to the fact that the volume sampled is smaller for region ***A*** than for region ***B***. There is some interconnectivity between the voids, as can be deduced from the much larger diameters present in the *v_connect* graphs (top and middle panels).

Figure S12 highlights the most prominent MOF/polymer interactions, by radial distribution functions analyses.

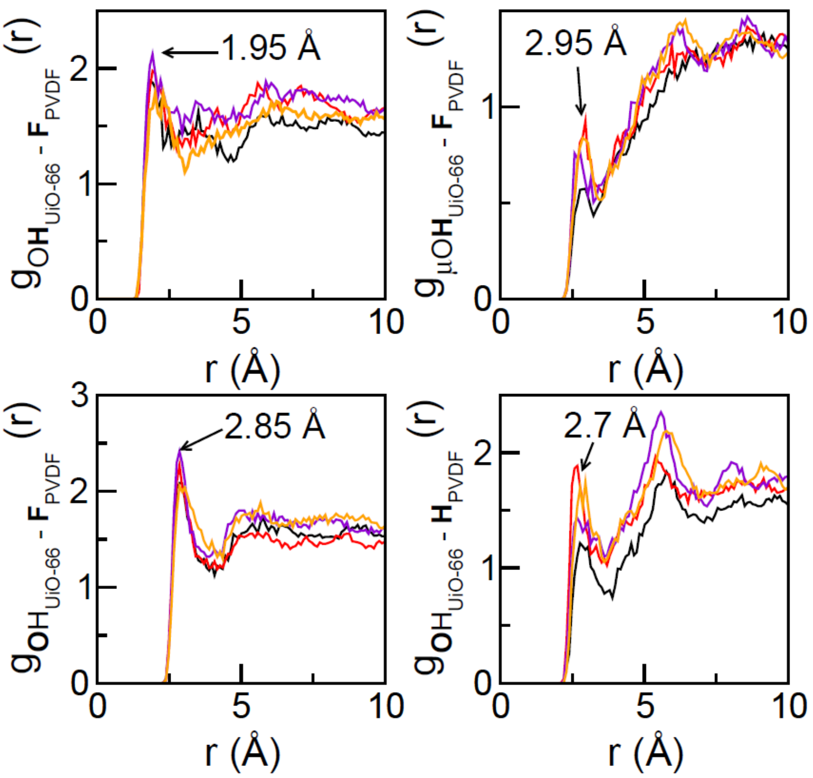

Figure S12. Radial distribution functions between UiO-66 terminations and PVDF F and H sites. The different colored curves represent results from different molecular dynamics simulations.

Several MOF/polymer site-site interactions can be identified in these composites. For UiO-66/PVDF, the main interaction is the H bond formed by the OH_{UiO-66} terminations (atom type H4 in Figure S1) with the F_{PVDF} atoms, with a characteristic length of 1.95 Å (see left upper panel in Figure S12). Weaker interactions can also be found between these terminations (atom type O4) and the H_{PVDF}, and between the F_{PVDF} sites and the H_{UiO-66} (atom type H3) associated to the μOH_{UiO-66}, with distances of 2.7 and 2.95 Å respectively.

<u>UiO-66/PEG interface</u>

The behavior of the density profile for this interface is analogue to that described for the UiO-66/PVDF interface in the manuscript. MOF and polymer atoms coexist throughout all region ***A*** (see Figure S13).

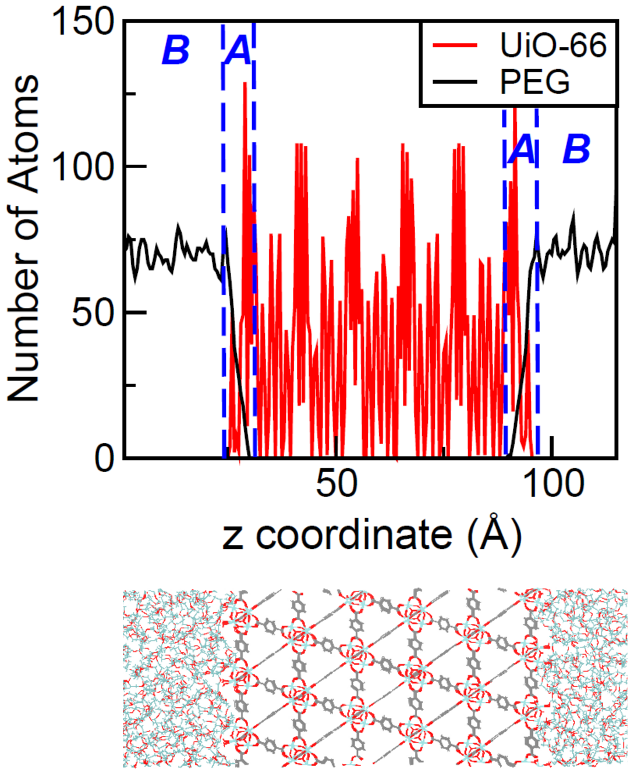

Figure S13. Atomic density of PEG (black) and UiO-66 (red) in the direction perpendicular to the surface slab. Note the MOF/polymer overlap in region ***A***. A scheme for the interface is also provided (color code as in Figures S1 and S5).

The polymer porosity is similar in region ***A*** and ***B***, with maximum pore diameters of 4 Å, as shown in Figure S14, bottom panels (pore size distribution). These pores are interconnected, which can be read from the fact that larger values are obtained from the *v_connect* analysis.

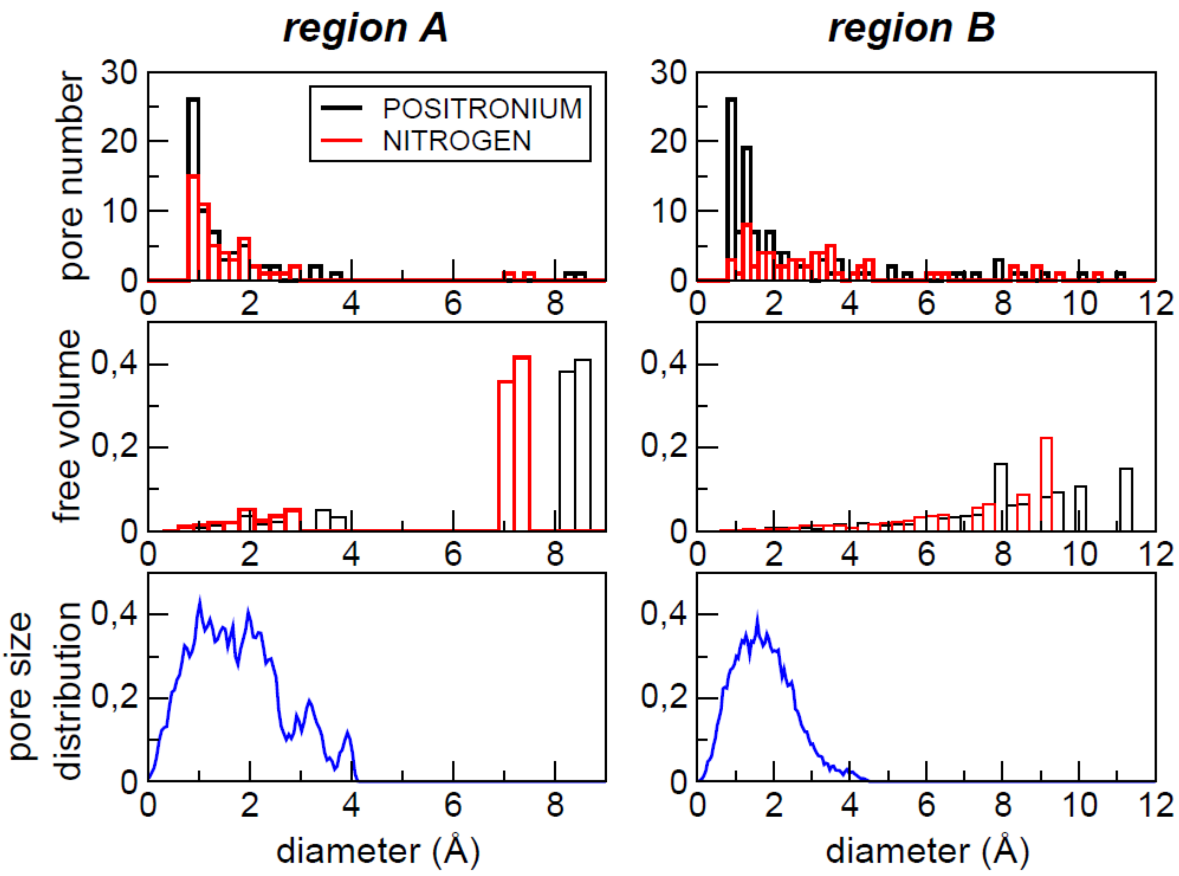

Figure S14. Histograms characterizing the sizes of the voids in region ***A*** (left) and region ***B*** (right) for a representative configuration of the model UiO-66/PEG interface. Number of pores and free volume fraction (top and middle) as a function of the diameter of the void. Bottom: pore size distribution.

Our computational analysis finishes with an exploration of the interactions that hold the UiO-66 surface together with the PEG phase, as shown in Figure S15.

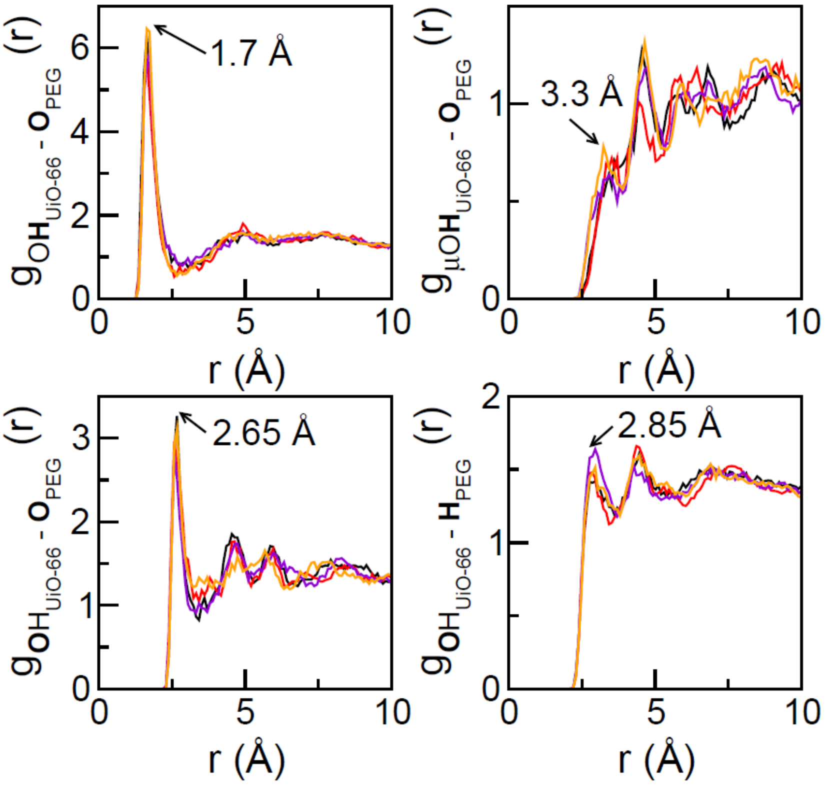

Figure S15. Radial distribution functions between UiO-66 terminations and PEG sites. The different colored curves represent results from different molecular dynamics simulations.

A much shorter and three-fold intense peak at 1.7 Å in the top left panel indicates the strongest site-site interaction observed for the composites studied, a hydrogen bond between the O_{PEG} and the OH_{UiO-66} terminations (atom type H4 in Figure S1). The interaction with the μOH_{UiO-66} corresponds to much longer distance of 3.3 Å. H_{PEG} interacts with the O_{UiO-66} in the OH_{UiO-66} terminations.

Since this MOF/polymer pair exhibits a very good coverage, we additionally studied the interactions with the organic linkers, these are plotted in Figure S16. There are interactions of similar lengths as some of those found for the terminations, the H_{PEG} sites interact with the aromatic H_{UiO-66} and with the COO_{UiO66} (atom types H1 and C1 in Figure S1) with characteristic distances of 2.8 and 3.3 Å respectively. The intensities are comparatively low because all of the organic linkers were considered in the analysis, and not only those at the surface. It can be concluded that the MOF/polymer interactions are more or less uniform all along the *xy* plane of the surface, and this contributes to explain the good adhesion and thus the pore blockage observed experimentally.

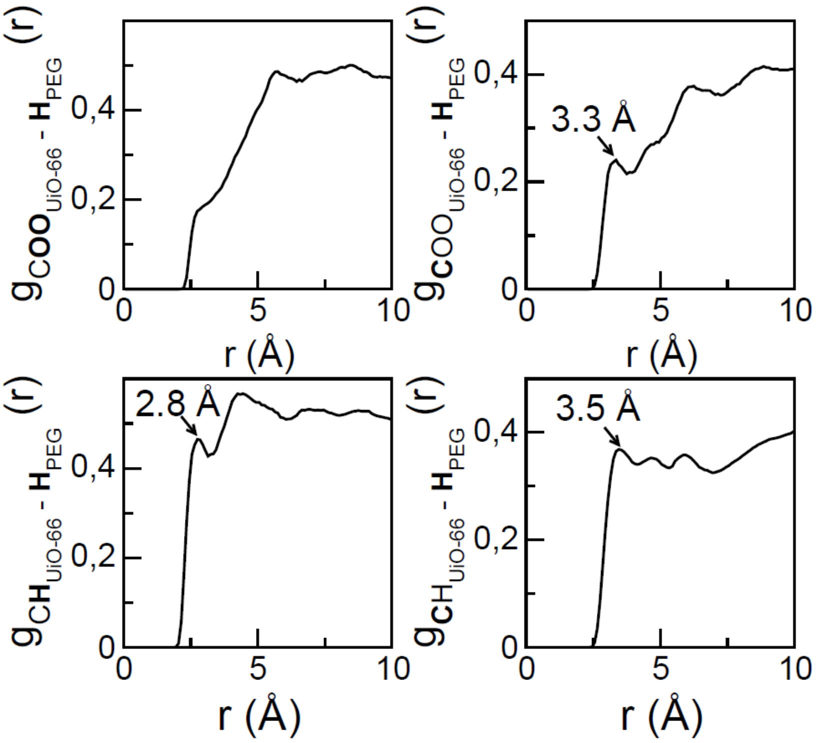

Figure S16. Radial distribution functions between UiO-66 organic linkers and PEG sites. The different colored curves represent results from different molecular dynamics simulations.

C) EXPERIMENTAL DETAILS

Synthetic Procedures

Synthesis of UiO-66: Zirconium(IV) chloride (61 mg, 0.26 mmol) and terephthalic acid (43 mg, 0.26 mmol) were dissolved in 15 mL DMF with 0.45 mL glacial acetic acid in a 20 mL vial with Teflon-lined cap. The vial was then placed in a 120 °C oven for 24 h. After cooling to ambient temperature, the particles were collected by centrifugation (fixed-angle rotor, 6000 rpm, 10 min), followed by washing with 3×10 mL DMF and 3×10 mL MeOH. The particles were then soaked in MeOH for 3 d, with solvent changed daily, before being dried under vacuum at room temperature and weighed. Yield: 55 mg (76%).

PVDF MMM Fabrication: UiO-66 was synthesized according to the procedure reported above, then dispersed in acetone (3.5 wt% MOF) via sonication. PVDF (M_w = 750,000 g/mol, PDI = 2.2; polymer and characterization data obtained from Arkema) was dissolved in DMF to a honey-like viscosity (7 wt%) then the MOF and polymer solutions were mixed to generate a 70 wt% MOF/ 30 wt% PVDF mixture, and ultrasonicated to homogeneity. Using the draw-down method, the MOF/PVDF solution was transferred to an aluminum foil substrate and then cast with a MTI Corporation MSK-AFA-II automatic thick film coater using an adjustable doctor blade set to 400 μm, at a speed of 25 mm/second. The as-cast films were then oven-cured at 70 °C until dry (roughly 1h) and the aluminum backing was peeled away with tweezers. Most MMMs were composed of a total of 200-500 mg of combined MOF and polymer components.

Materials Characterization

Powder X-ray Diffraction (PXRD): PXRD data was collected at room temperature on a Bruker D8 Advance diffractometer, running at 40 kV, 4 mA for Cu Kα (λ = 1.5418 Å), with a

scan speed of 0.5 sec/step, a step size of 0.02° in 2θ, and a 2θ range of 5-50°. Sample holders used were zero-background Si plates (p-type, B-doped) from MTI Corp. Well-type sample holders (0.5 mm depth) were used for powder samples. MMM and pure-polymer samples were affixed to flat sample holders using double-sided Scotch tape prior to measurement. Due to differences in sample preparation between the UiO-66 powder and the PEG-based MMMs, direct comparison of absolute peak intensity differences is not possible.[12]

BET Analysis of N_2 Sorption Isotherms: N_2 sorption isotherms were collected as described in the manuscript. BET internal surface areas were then determined from analysis of the Rouquerol[13] plots of the isotherm data, using 4-10 data points each. The guidelines set forth by Rouquerol[13] use four criteria to obtain the most accurate BET surface area values for microporous materials such as MOFs. Further work by Snurr,[14] specific to UiO-66, recommends the implementation of criteria I-III to obtain the most accurate BET area measurement for this specific material (since criteria IV is not met in UiO-66).[14] Criteria I, that BET constant C must be positive, and criteria II, that the value $V(1\text{-}p/p_0)$ must increase with increasing p/p_0 for all points chosen, are both true for the UiO-66 and MMMs in this study. Similarly, criteria III states that the total monolayer loading should correspond to a relative pressure within the selected linear region, and holds true for our measurements on UiO-66 and all MMMs.

Scanning Electron Microscopy (SEM): MOF and MMM samples were placed on conductive carbon tape on a sample holder and coated using an Ir sputter-coating for 7 s. A Phillips XL ESEM microscope was used for acquiring images using a 15 kV energy source under vacuum at a working distance of 10 mm.

TGA Analysis: MOF and MMM samples (~5-10mg) were weighed and placed in alumina crucibles. Samples were analyzed under a flow of dry N_2 gas at a flow rate of 70 mL/min from 30 °C to 800 °C at a heating rate of 5 °C/min on a Mettler Toledo TGA/DSC 1 STARe system.

DSC Analysis: MOF and MMM samples (~5-10mg) were weighed and placed in alumina crucibles. Samples were analyzed under a flow of dry N_2 gas at a flow rate of 70 mL/min on a Mettler Toledo TGA/DSC 1 STARe system. PVDF-based samples were first heated to 200 °C to remove the thermal history, then cooled to 100 °C, then heated again to 200 °C, all at a rate of 10 °C/min. PEG-based samples were first heated to 80 °C at 10 °C/min to remove the thermal history, then cooled at 10 °C/min to 30 °C. The sample was then held at 30 °C for 10 min to stabilize the temperature reading, then heated to 80 °C at 5 °C/min to maximize the resolution of the melt event.

D) ADDITIONAL EXPERIMENTAL RESULTS

Table S7. BET area measurements and constants are given below for all MMMs tested. All reported BET surface area values are the average of at least 3 independent samples, while BET constants C and Q_m are representative values from one sample.

Polymer	*% MOF*	*BET Surface Area*	*BET Constant C*	*BET Constant Q_m*
--	100	1382 ± 58	2122	331
PVDF	70	759±66	1472	185
PEG	70	29 ± 6	69	6
	80	480 ± 50	3410	116

Table S8. Ultimate tensile strength and Young's modulus values for all films tested. All values are the average of at least five independent measurements.

Sample	*Ultimate tensile strength (MPa)*	*Young's modulus (MPa)*
PVDF	29.7 ± 3.8	802 ± 66
PVDF MMM	3.6 ± 0.5	770 ± 54
PEG	3.3 ± 0.9	133 ± 13
PEG MMM	1.1 ± 0.1	284 ± 124

a) b)

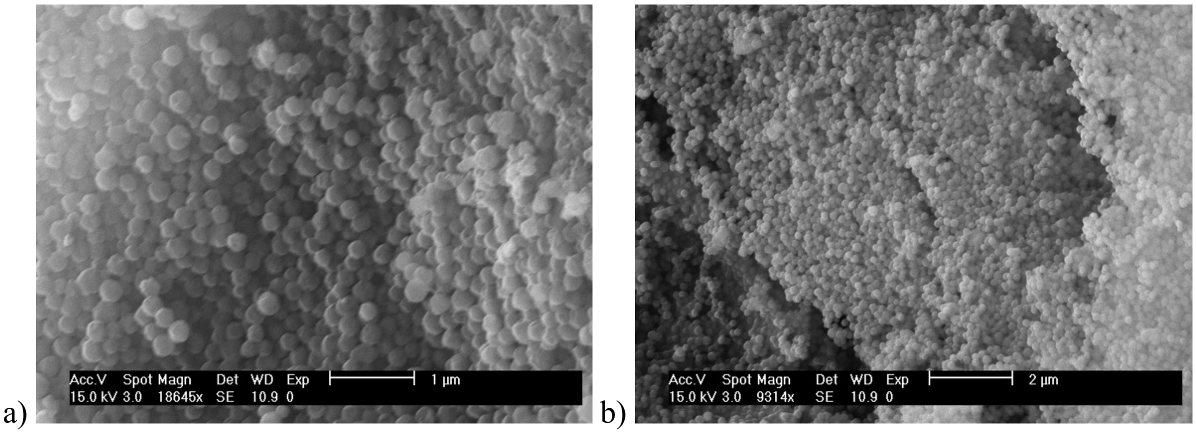

Figure S17. SEM images of as-synthesized UiO-66 at two different magnifications (scale bars of a) 1 µm, b) 2 µm). These images highlight the uniform ~200 nm diameter size and roughly spherical morphology of the particles.

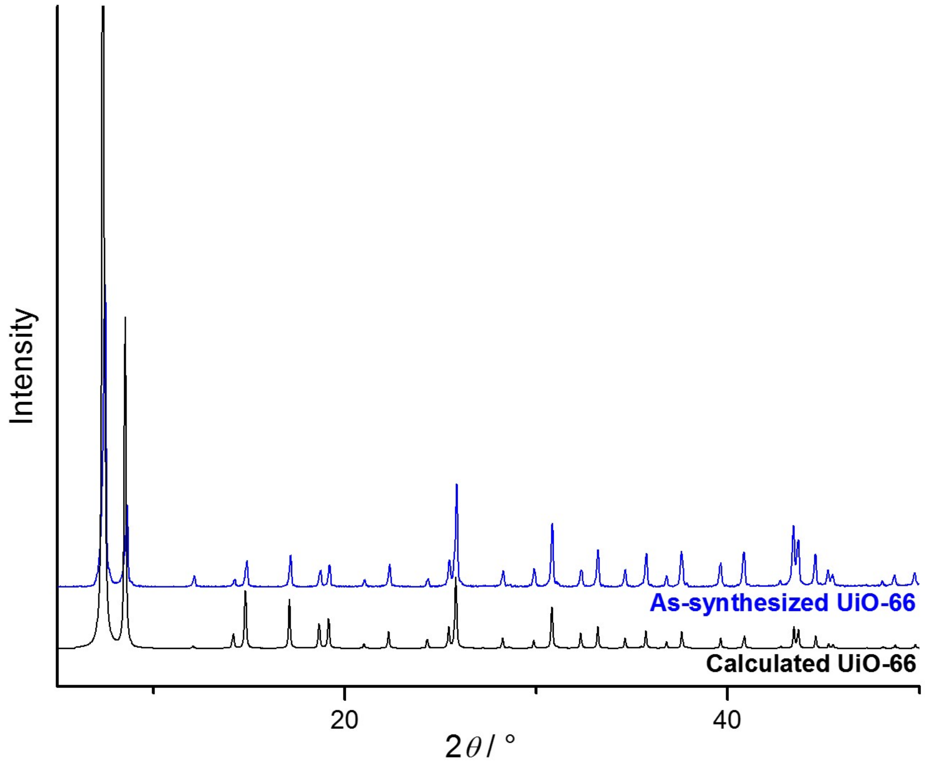

Figure S18. PXRD of as-synthesized UiO-66 used in this study with the calculated powder pattern for comparison.

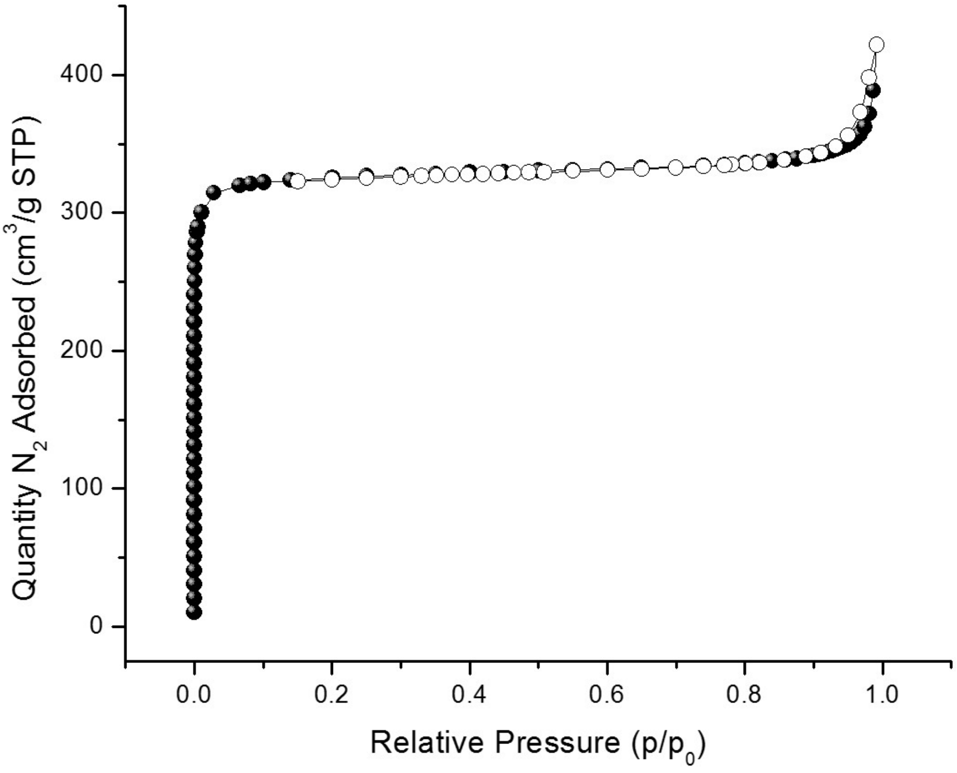

Figure S19. Nitrogen sorption isotherm data of as-synthesized UiO-66 used in this study.

a) 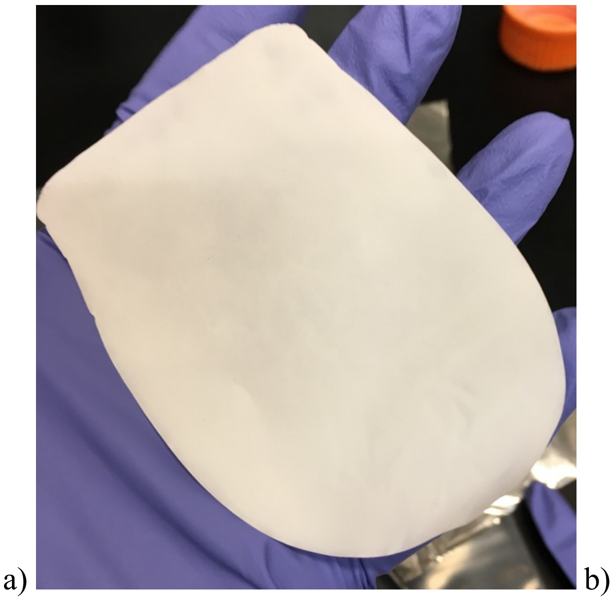 b) 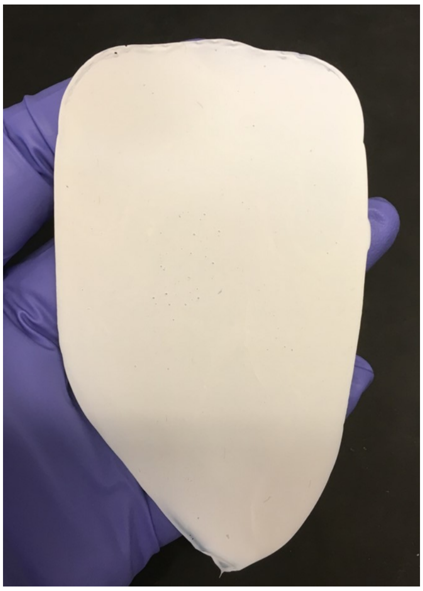

Figure S20. Full-size MMMs at a) 70 wt% MOF and b) 80 wt% MOF in PEG demonstrate that they are continuous, uniform and easily delaminated from the Al foil substrate.

a) 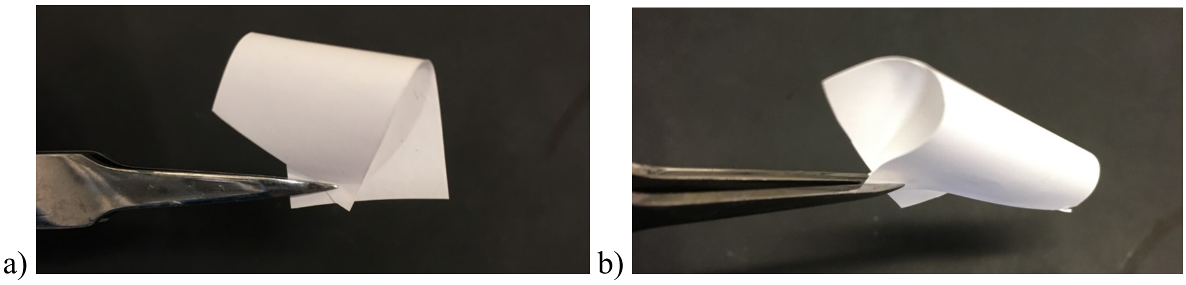 b)

Figure S21. Sections of the above MMMs are cut and bent, showing their flexibility at both a) 70 wt% MOF and b) 80 wt% MOF in PEG.

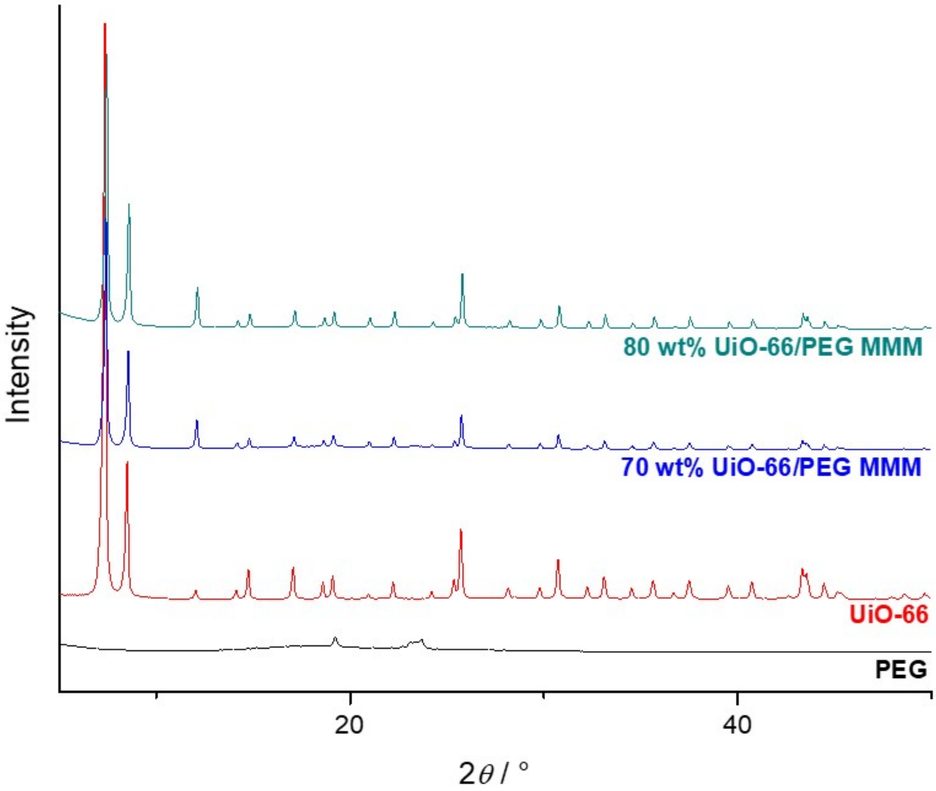

Figure S22. PXRD patterns of PEG MMMs compared to the UiO-66 and PEG starting materials. The polymer alone displays mostly amorphous character, with small features at 19° and 23°. The MMMs show the same powder pattern as UiO-66 alone, demonstrating that the MOF remains highly crystalline within the MMM.

a) 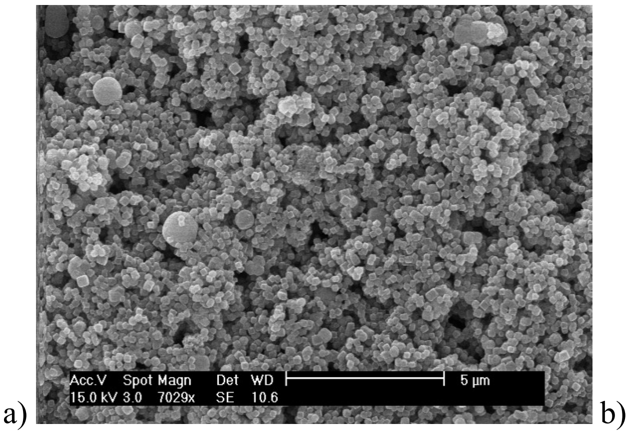

b) 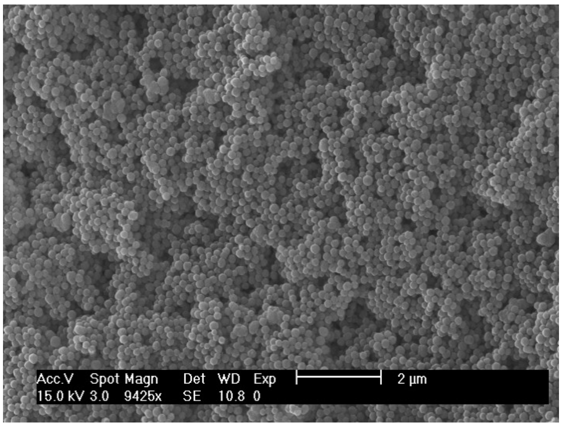

Figure S23. Comparison of top-view SEM images of PEG-based MMMs at a) 70 wt% MOF and b) 80 wt% MOF. No significant differences can be seen, and the PEG component is not visible in either image. Scale bars are 2µm.

a) 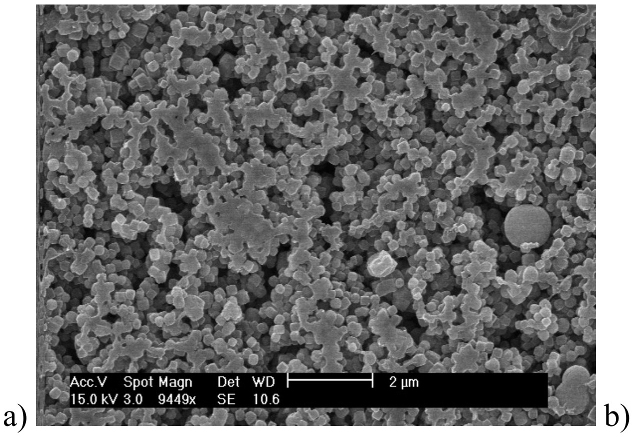

b) 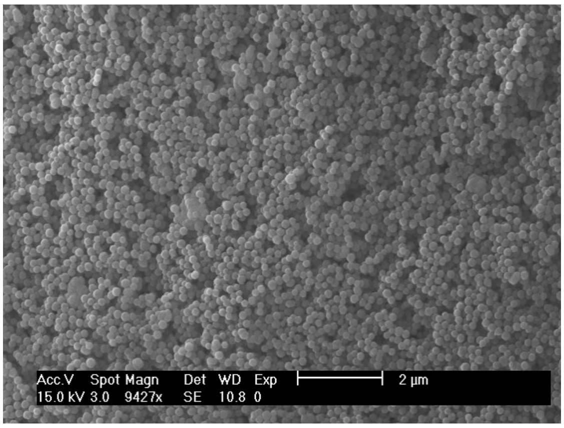

Figure S24. Comparison of SEM images of the Al-foil side of PEG-based MMMs at a) 70 wt% MOF and b) 80 wt% MOF. The polymer component is visible in the 70 wt% MMM in the continuous, flattened regions while the 80 wt% MMM shows no evidence of polymer component. Scale bars are 5 and 2µm for (a) and (b), respectively.

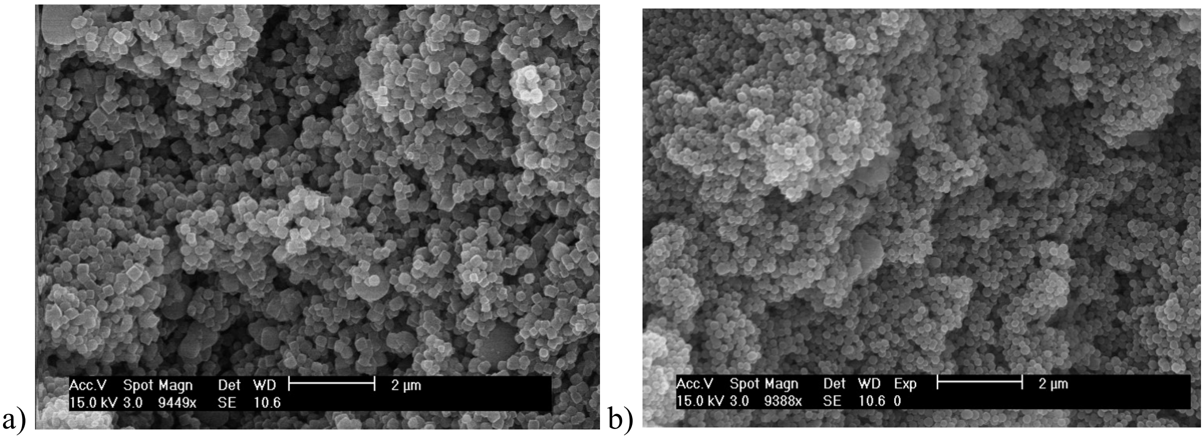

Figure S25. Comparison of SEM images of the cross-section of PEG-based MMMs at a) 70 wt% MOF and b) 80 wt% MOF. The polymer component is not visible in either MMM, and both exhibit a MOF-dominant, open morphology on the interior of the MMM. Scale bars are 2µm.

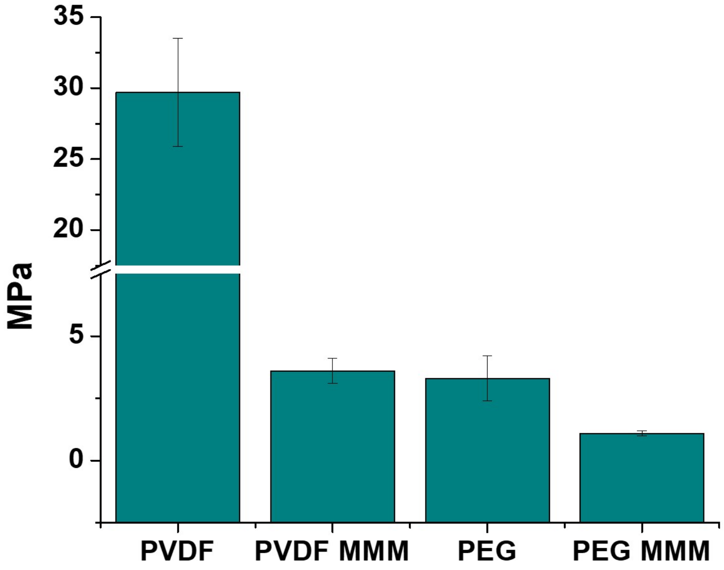

Figure S26. Ultimate tensile strength measurements on pure polymer and 70 wt% MOF MMMs. Pure PVDF is by far the strongest material, with similar values measured for the MMMs and pure PEG.

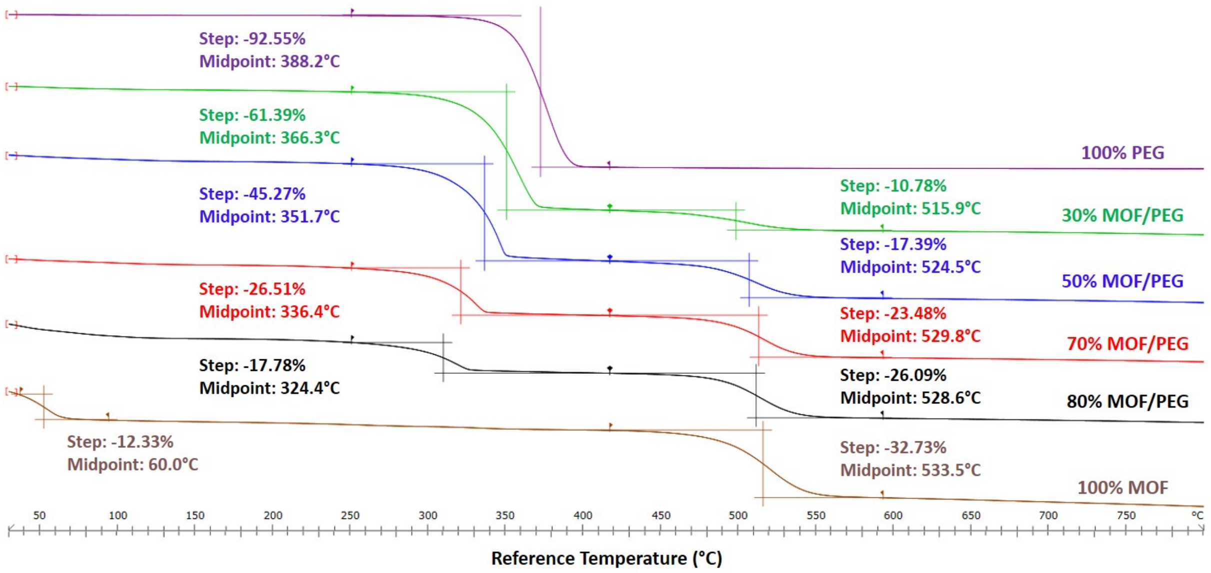

Figure S27. TGA data of PEG and PEG-based MMMs. Data is staggered on y-axis to emphasize differences in degradation temperature (T_d) of polymer.

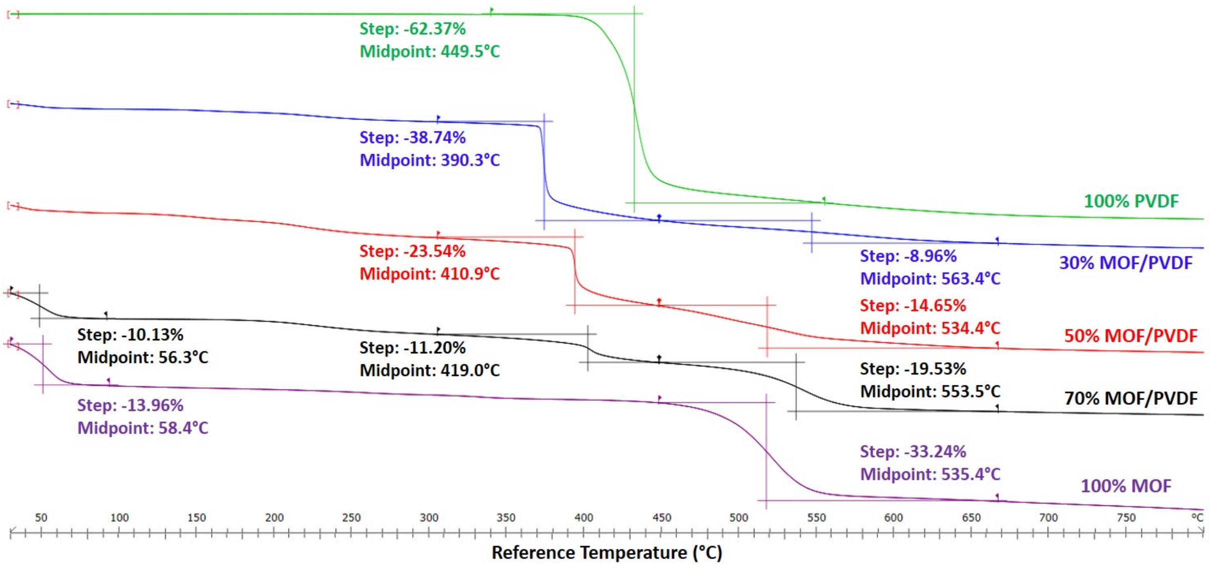

Figure S28. TGA data of PVDF and PVDF-based MMMs. Data is staggered on y-axis to emphasize differences in T_d of polymer.

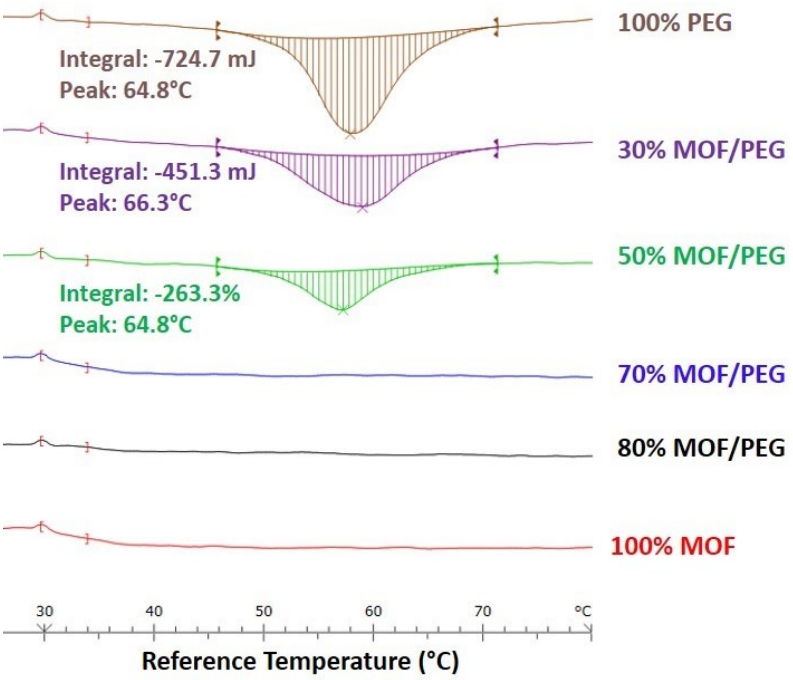

Figure S29. DSC traces of PEG and PEG-based MMMs. Endotherms are indicated as negative, and shaded portions represent melt events of polymer. Data is staggered on y-axis to emphasize differences in melting temperature (T_m) of polymer. Peak temperatures are actual sample temperature, and may not directly correlate with x-axis (reference temperature).

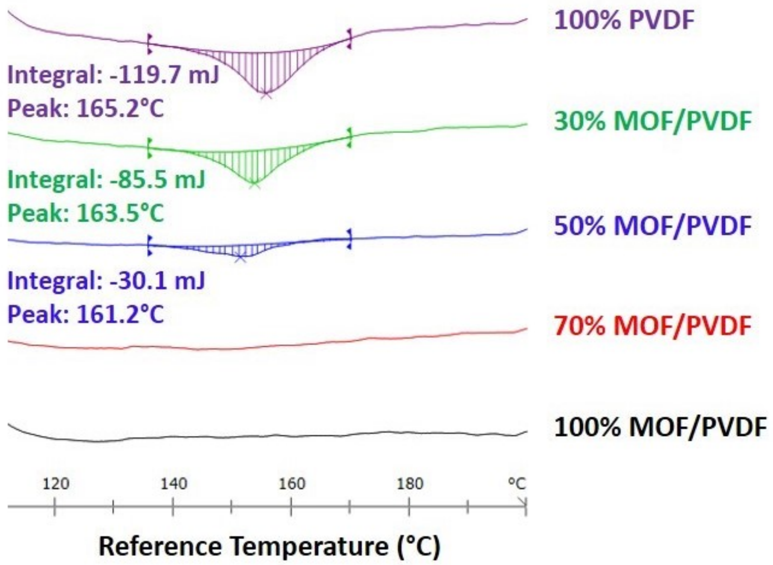

Figure S30. DSC traces of PVDF and PVDF-based MMMs. Endotherms are indicated as negative, and shaded portions represent melt events of polymer. Data is staggered on y-axis to emphasize differences in T_m of polymer. Peak temperatures are actual sample temperature, and may not directly correlate with x-axis (reference temperature).

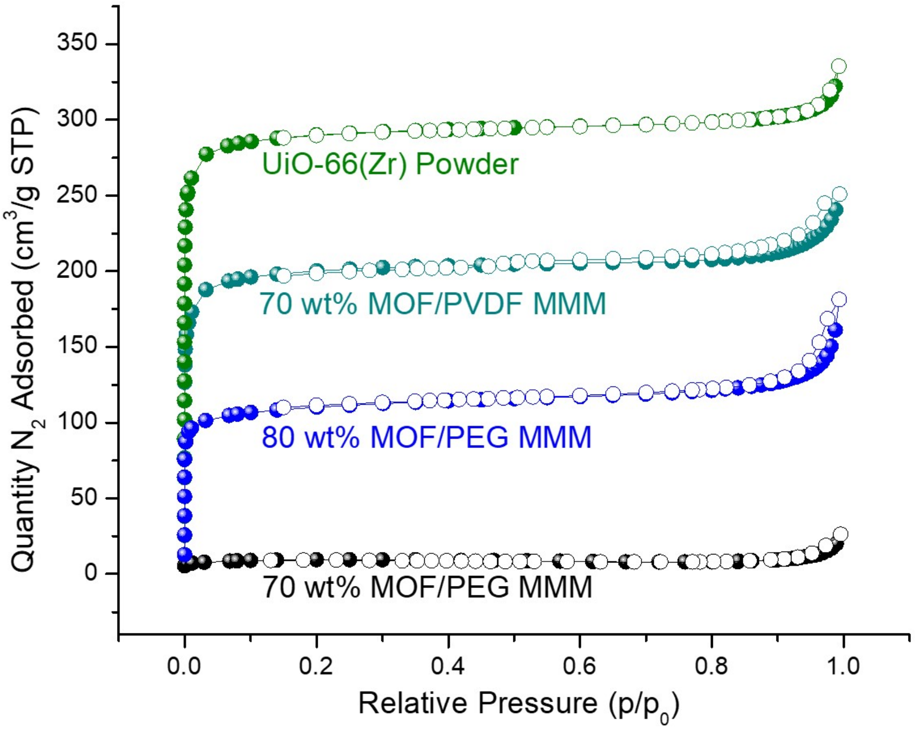

Figure S31. Nitrogen sorption isotherms collected at 77 K of MOF powder and various MMMs, labeled accordingly.

References

1 Q. Yang, H. Jobic, F. Salles, D. Kolokolov, V. Guillerm, C. Serre and G. Maurin, *Chem. - A Eur. J.*, 2011, **17**, 8882–8889.

2 B. L. Eggimann, A. J. Sunnarborg, H. D. Stern, A. P. Bliss and J. I. Siepmann, *Mol. Simul.*, 2014, **40**, 101–105.

3 R. Semino, N. A. Ramsahye, A. Ghoufi and G. Maurin, *ACS Appl. Mater. Interfaces*, 2016, **8**, 809–819.

4 S. L. Mayo, B. D. Olafson and W. A. Goddard, *J. Phys. Chem.*, 1990, **94**, 8897–8909.

5 J. P. Perdew, K. Burke and M. Ernzerhof, *Phys. Rev. Lett.*, 1996, **77**, 3865–3868.

6 W. J. Hehre, R. Ditchfield and J. A. Pople, *J. Chem. Phys.*, 1972, **56**, 2257–2261.

7 B. Delley, *J. Chem. Phys.*, 1990, **92**, 508–517.

8 *Materials Studio*, Accelrys Software Inc., 2001.

9 J. Wang, R. M. Wolf, J. W. Caldwell, P. A. Kollman and D. A. Case, *J. Comput. Chem.*, 2004, **25**, 1157–1174.

10 D. Hofmann, M. Heuchel, Y. Yampolskii, V. Khotimskii and V. Shantarovich,

Macromolecules, 2002, **35**, 2129–2140.

11 S. Bhattacharya and K. E. Gubbins, *Langmuir*, 2006, **22**, 7726–7731.

12 T. Uemura, N. Yanai, S. Watanabe, H. Tanaka, R. Numaguchi, M. T. Miyahara, Y. Ohta, M. Nagaoka and S. Kitagawa, *Nat. Commun.*, 2010, **1**, 1–8.

13 J. Rouquerol, P. Llewellyn and F. Rouquerol, *Studies in Surface Science and Catalysis*, Elsevier, 2007.

14 D. A. Gómez-Gualdrón, P. Z. Moghadam, J. T. Hupp, O. K. Farha and R. Q. Snurr, *J. Am. Chem. Soc.*, 2016, **138**, 215–224.
